# Performance of clinical decision aids (CDA) for the care of young febrile infants: a multicentre prospective cohort study conducted in the UK and Ireland

**DOI:** 10.1016/j.eclinm.2024.102961

**Published:** 2024-11-27

**Authors:** Etimbuk Umana, Clare Mills, Hannah Norman–Bruce, Hannah Mitchell, Lisa McFetridge, Fiona Lynn, Gareth McKeeman, Steven Foster, Michael J. Barrett, Damian Roland, Mark D. Lyttle, Chris Watson, Thomas Waterfield, Paediatric Emergency Research in the UK and Ireland (PERUKI)

**Affiliations:** a Wellcome Wolfson Institute of Experimental Medicine, Queen's University Belfast, Belfast, UK; b Mathematical Sciences Research Centre, Queen's University Belfast, Belfast, UK; c School of Nursing and Midwifery, Queen's University Belfast, Belfast, UK; d Department of Clinical Biochemistry, Belfast Health and Social Care Trust, Belfast, UK; e Emergency Department, Royal Hospital for Children, Glasgow, UK; f Paediatric Emergency Medicine, Children's Health Ireland at Crumlin, Dublin, Ireland; g Women's and Children's Health, School of Medicine, University College Dublin, Ireland; h Sapphire Group, Health Sciences, University of Leicester, Leicester, UK; i Paediatric Emergency Medicine Leicester Academic (PEMLA) Group, University Hospitals of Leicester NHS Trust Leicester, UK; j Emergency Department, Bristol Royal Hospital for Children, Bristol, UK; k Faculty of Health and Applied Sciences, University of the West of England, Bristol, UK; l Emergency Department, Royal Belfast Hospital for Sick Children, Belfast, UK

**Keywords:** Invasive bacterial infection, Clinical decision aid, Febrile infant

## Abstract

**Background:**

Between 1% and 4% of febrile infants, aged from birth to 90 days of age, presenting to hospital will be diagnosed with an invasive bacterial infection (IBI). Traditional teaching has advocated a treat all approach but more recently a number of clinical decision aids (CDA) have been developed to classify febrile infants into lower and higher risk cohorts, with lower risk infants suitable for management without immediate parenteral antibiotics and lumbar puncture. The aim of this study was to apply these CDA to a UK and Irish cohort.

**Methods:**

This was a prospective multicentre cohort study of febrile infants presenting to 35 Paediatric Emergency Research in the UK and Ireland (PERUKI) sites between the 6th July 2022 and the 31st August 2023. All infants received standard care as per local policy. IBI was defined as growth of bacterial pathogen in blood or cerebrospinal fluid. The performance of the following CDAs were assessed, National Institute for Health and Care Excellence (NICE) guidelines NG143 (Fever under 5 years), British Society Antimicrobial Chemotherapy (BSAC), Aronson rule and American Academy of Pediatrics (AAP) CDA. A cost comparison of each CDA against a treat all approach was conducted. Trial registration: NCT05259683.

**Findings:**

1821 were included in the final analysis. The median age was 46 days (IQR: 30–64 days), with 1108 (61%) being male. Of the 1821 infants, 67 (3.7%) had IBI. The AAP and BSAC CDAs were the most sensitive at 0.99 (95% CI 0.92–1.0) for both with specificities of 0.23 (95% CI 0.21–0.25) and 0.20 (95% CI 0.18–0.22) respectively. The NICE NG143 and Aronson CDA were the most specific CDAs with values of 0.27 (95% CI 0.25–0.30) and 0.30 (95% CI 0.28–0.32) respectively, but their sensitivity was lower. The AAP CDA performed equally well with either procalcitonin (PCT) or C-reactive protein (CRP) as the biomarker of choice. Of the 1821 infants, 77% were admitted, 14% were discharged and 9% were ambulated. All CDAs were cost saving for hospital services when compared to a treat all approach, with the lowest mean cost per patient estimated for Aronson (£1171; bootstrap 95% CI £1129–£1214) and NICE NG143 CDA (£1218; bootstrap 95% CI £1174–£1263).

**Interpretation:**

The AAP and BSAC CDAs are highly sensitive at excluding IBI, with a cost saving to hospital services when compared to a treat all approach. The substitution of CRP for PCT made no difference to the performance of the AAP CDA in this cohort and was more costly.

**Funding:**

The Febrile Infant Diagnostic Assessment and Outcome (FIDO) study is funded by Royal College of Emergency Medicine Doctoral Fellowship (RCEM 02/03/2021). Procalcitonin analysis was supported by the Public Health Agency Northen Ireland Grant (HSC R&D-COM/5745/22). The funders played no part in the conception or design of this study.

**Research in context**

**Evidence before this study**

Managing young febrile infants is challenging for clinicians, with 1–4% at risk of invasive bacterial infection (IBI). Clinical decision aids (CDAs), such as the National Institute for Health and Care Excellence (NICE) NG143, British Society for Antimicrobial Chemotherapy (BSAC), Aronson rule, and the American Academy of Pediatrics (AAP) guidance for febrile infants have been validated in a range of international settings and used to identify a low risk cohort for tailored management. These CDAs have not been prospectively validated in the UK and Ireland and it is unclear if the AAP CDA can safely be applied in the UK and Ireland without procalcitonin (PCT) testing.

**Added value of this study**

In this large prospective multicentre study, the NICE NG143, BSAC, Aronson and AAP CDA (with CRP or PCT) were validated in 35 UK and Irish hospitals. The AAP and BSAC were the best performing CDAs identifying low risk cohorts of 22% and 20% respectively that could be managed without lumbar puncture or parenteral antibiotics. There was no difference in performance of the AAP CDA with either CRP or PCT as the biomarker of choice.

**Implications of all the available evidence**

This study supports the use of tailored CDAs such as the AAP and BSAC for the risk stratification of febrile infants in the UK and Ireland. Furthermore, AAP guidance can be applied safely in settings without access to PCT testing. This could lead to decreased lumbar puncture rates, improved antimicrobial stewardship and lower treatment costs.

## Introduction

Young infants (aged 0 and 90 days) with a fever are at high risk of having bacterial meningitis and bacteraemia, collectively known as invasive bacterial infection (IBI).^1^ The prevalence of IBI in this population ranges between 1 and 4%.^2^, ^3^, ^4^ Prompt recognition of IBI in this cohort is likely to lead to better outcomes, therefore a treat all approach has been traditionally advised.^5^ Over the last 30 years, this has been challenged with a move towards tailored care based on sequential assessment.^6^ Advocates of this approach cite changes in the epidemiology and aetiology of IBI over time as key contributing factors to the safety of this approach.^1^^,^^7^^,^^8^ Introduction of maternal and infant vaccines combined with public health measures and maternal health screening have reduced the burden of *Group B streptococcus* and *Listeria* infection in this cohort with a shift towards gram negative organisms such as *Escherichia coli*.^6^^,^^9^, ^10^, ^11^ Reported benefits of tailored care include shorter hospital stays, fewer invasive procedures, reduced admission rates which result in reduced health care costs, and better antimicrobial stewardship.^12^^,^^13^

Internationally a range of clinical decision aids (CDA) exist to guide the management of young febrile infants. In the UK this includes the National Institute for Health and Care Excellence (NICE) guideline NG143 (Fever under 5 years) and the British Society for Antimicrobial Chemotherapy (BSAC) CDA.^14^^,^^15^ In North America and Europe, CDAs including StepByStep,^3^ Pediatric Emergency Care Applied Research Network (PECARN),^2^ Aronson rule,^16^ and The American Academy of Pediatrics (AAP) CDAs have been developed.^6^ All utilise sequential assessment based on age, clinical appearance and biomarker testing to identify a lower risk cohort suitable in whom lumbar puncture and parenteral antibiotics can be averted. Validation studies of these CDAs vary by inclusion criteria and methodology, but typically report sensitivities between 92% and 100% for excluding IBI.^17^, ^18^, ^19^, ^20^, ^21^, ^22^, ^23^

The CDAs with the most robust validation data (StepByStep, PECARN, and AAP) require procalcitonin (PCT) testing as part of the sequential assessment.^2^^,^^3^^,^^6^ The use of PCT in this cohort is supported by a systematic review and meta-analysis (14 studies; 7755 young febrile infants) which demonstrated that a PCT value of 0.5 ng/ml has a greater partial area under the curve (pAUC 0.72; 95% CI 0.56–0.79) than a C-reactive protein (CRP) value of 20 mg/L (pAUC 0.28; 95% CI 0.17–0.61), p = 0.016 for detecting IBI.^24^ In settings without access to PCT testing it remains unclear whether sequential assessment can safely identify a cohort of young febrile infants who can be managed without parenteral antibiotics. Of all the available CDAs only the AAP provides guidance on the use of CRP where PCT is unavailable.^6^

In the Febrile Infant Diagnostic Assessment and Outcome (FIDO) Study we aimed to prospectively assess the management of young febrile infants presenting to emergency care in the UK and Ireland, report risk factors for IBI in this population, and apply CDAs that do not exclusively rely on PCT testing.

## Methods

### Study design and participants

This prospective multicentre cohort was conducted at 35 paediatric emergency departments (ED) and paediatric assessment units (AU) between the 6th July 2022 and 31st August 2023. The study protocol has been previously published and adheres to TRIPOD, STARD and CHEERS statements.^21^^,^^25^, ^26^, ^27^ Participating centres were distributed across the UK and Ireland with one in Northern Ireland, one in the Republic of Ireland, one in Wales, two in Scotland, and 30 in England. Sites included tertiary paediatric specialist and district general hospitals; a summary of sites characteristics and recruitment can be found in the Supplementary Table S1. Infants aged 90 days or younger with a fever ≥38 °C in the ED/AU, or with a history of fever ≥38 °C recorded by anyone via any thermometer within 24 h before presentation, were eligible for inclusion. Infants whose guardians declined or withdrew consent were excluded. Table 1 shows the characteristics of the FIDO cohort.

**Table 1:** Patient characteristics comparing total population and PCT population.

Variable	Total n-1821	Non PCT group n-1355 (%)	PCT group n-466 (%)	p value
**Age category (28 day)**				0.906
<28 days	434 (23.8)	322 (23.8)	112 (24.0)	
>/= 29 days	1387 (76.2)	1033 (76.2)	354 (76.0)	
**Gender**				0.704
Male	1108 (60.8)	821 (60.6)	287 (61.6)	
Female	713 (39.2)	534 (39.4)	179 (38.4)	
**Comorbidities present**	260 (14.3)	191 (14.1)	69 (14.8)	0.705
**Presenting < 6 h from fever onset**	966 (53.0)	719 (53.1)	247 (53.0)	0.332
**Fever with source**	1029 (56.5)	770 (56.8)	259 (55.6)	0.639
**Temperature**	38.0 (37.4–38.4)	38.0 (37.3–38.4)	38.0 (37.4–38.4)	0.243
**Unwell appearing**	1058 (58.1)	750 (55.4)	308 (66.1)	<0.001
**Antibiotics Administered**	1242 (68.2)	885 (65.3)	357 (76.6)	<0.001
**Dispositions**				<0.001
Discharged from ED	247 (13.6)	216 (15.9)	31 (6.7)	
Ambulated	167 (9.2)	113 (8.3)	54 (11.6)	
Admitted	1407 (77.2)	1026 (75.7)	381 (81.8)	

ED, Emergency department; PCT, Procalcitonin.

### Outcome measures

The primary outcome measure was the performance accuracy of CDAs at identifying children with IBI. Secondary outcome measures included aetiology of IBI, clinical predictors of IBI and the mean cost per patient for each CDA.

### Identifying clinical risk factors

Potential clinical risk factors for IBI were identified through a review of the literature and by reviewing CDAs from all participating sites.^28^ The identified risk factors were then included in the case report form (CRF) (Supplementary Figures S1 and S2). Four CDAs were selected (NICE NG143, BSAC, Aronson and AAP) as they could be readily applied to settings without PCT testing. These CDAs and their low-risk criteria are described in Table 2. The clinical risk factors of IBI identified included background information (e.g. age, comorbidities), time to presentation from fever onset, unwell contacts, clinical appearance, vital signs, symptoms on presentation (e.g. cough, vomiting, diarrhoea), and clinical exam findings. Study definitions and CDA application for the FIDO study can be found in Supplementary material.

**Table 2:** CDAs and low risk criteria, year of derivation and derivation methodology.

CDA	Low risk criteria	Year of derivation	Derivation methodology
NICE NG143	Age over 1 month, well appearing, normal white cell count	2019	Expert consensus
Aronson rule	A score-based system including age, height of fever, urinalysis results and absolute neutrophil count. Score <2 classified as low risk	2019	Regression analysis
BSAC	Age over 1 month, well appearing, negative urinalysis, CRP less than 20 mg/L and a normal absolute neutrophil count	2020	Expert consensus
AAP-CRP	Age over 21 days, well appearing, CRP less than 20 mg/L, temperature <38.5 C and a normal absolute neutrophil count	2021	Expert consensus
AAP-PCT	Age over 21 days, well appearing, PCT less than 0.5 ng/ml, and a normal absolute neutrophil count	2021	Expert consensus

AAP, American Academy of Pediatrics; BSAC, British Society for Antimicrobial Chemotherapy; CDA, Clinical decision aid; CRP, C-reactive protein; IBI, invasive bacterial infection; NICE, National Institute for Health and Care Excellence; PCT, Procalcitonin.

### Study procedures

Patients were screened for eligibility by clinical staff at the time of initial assessment, and clinical findings were reported prospectively using an electronic CRF Supplementary Figures S1 and S2). These data were recorded contemporaneously by clinical staff prior to consent discussions, and before laboratory test results were available. All infants in the FIDO study received clinical care as per local guidance without delay. During routine care, where feasible, an additional 1 ml of blood was collected during the first episode of phlebotomy and stored as plasma for PCT testing^21^ (Supplementary material).

### Reference standards

The reference standard for identification of IBI was defined as positive culture or quantitative polymerase chain reaction (qPCR) for a bacterial pathogen from a sterile body site (eg. blood or cerebrospinal fluid (CSF)), performed by technicians blinded to clinical information at accredited National Health Service (NHS) hospital laboratories in the UK and Children Health Ireland laboratory in the Republic of Ireland. A priori list of pathogens and contaminants was employed for determining the reference standard (Supplementary Figure S3). Participants who did not have culture/qPCR testing were assumed not to have IBI provided that they were not subsequently found to have been diagnosed with IBI within seven days of discharge on checking hospital records.

### Consent model

Due to the need for emergency intervention in IBI, research without prior consent (RWPC) was used, described in the published protocol.^21^ Participants were enrolled prior to consent to ensure contemporaneous data collection; their parents/guardians were subsequently invited to provide consent at the earliest appropriate opportunity (typically within 24 h) once the infant’s clinical condition had stabilised.

### Data management

Study data were collected and managed using Research Electronic Data Capture (REDCap) tools.^29^^,^^30^ The initial CRF was completed by treating clinicians to contemporaneously record data regarding clinical features observed at the first clinician assessment. The second CRF was completed seven days after discharge and recorded investigation results, length of stay, and other aspects of care not susceptible to recall bias. Any infants with incomplete CRFs for study outcomes were excluded from the analysis. Prior to statistical analysis three authors (EU, LM, HM) checked the database for completeness of data. Two authors (EU and TW) applied the CDAs to the data set. As standard care was followed, not all infants underwent blood testing; where this was the case, data was assumed within the normal range (i.e. CRP <20 mg/l, white cell count (WCC) between 5∗10ˆ9/l and 15∗10ˆ9/l, Absolute Neutrophil Count (ANC) < 4.0∗10ˆ9/l) was undertaken. This approach was selected because no infants without blood testing were subsequently diagnosed with IBI. The population without blood testing results were those at lowest risk of IBI. To minimise potential bias from this approach the analysis was repeated (i) with incomplete biomarker data (CRP, WCC and ANC) imputed via imputation method with chained equations (MICE)^31^ to create an imputed dataset, and (ii) with cases with incomplete biomarker data (CRP, WCC and ANC) excluded.

### Statistics

The FIDO study population are described in terms of demographic characteristics, well/unwell appearance (infant were considered unwell appearing if they had an abnormal global assessment or abnormal vital signs at presentation), comorbidities (<37 weeks gestation, known maternal *Streptococcus agalactiae*, congenital heart disease, antibiotics within the last 48 h, indwelling device), risk factors for IBI, investigations performed, parenteral antibiotic use, admission to hospital, admission to intensive care units, and survival using descriptive statistics. CDA performance was compared using sensitivity, specificity, negative predictive value (NPV), and positive predictive value (PPV), with 95% confidence intervals. McNemar's test was used to assess the significance of differences in sensitivities and specificities between CDAs. Sensitivity analyses were performed for infants with comorbidities excluded. Infants were deemed alive if on review of clinical notes, the patient had not re-attended and died. Clinical risk factors were assessed in a stepwise approach. Initially all possible clinical predictors were assessed using univariate analysis with Chi-squared testing of categorical data, and the Mann–Whitney U test for continuous data (continuous data were skewed). Age-dependent predictors such as heart rate, respiratory rate and blood pressure were converted to categorical data and classified as normal or abnormal based on published normal ranges.^32^ A 2-sided p value of <0.05 was considered statistically significant. Predictors with a statistically significant association with IBI (p < 0.20) were included in a binary multivariate logistic regression model. This liberal level of significance (p < 0.20) was chosen to avoid falsely excluding significant variables based on univariate analysis alone. Clinical predictors identified from the univariate analysis were then included in logistic regression modelling. Empirical binary multivariable forward and backward logistic regression modeling was used to identify a best-fit model to identify children at lowest risk of IBI. Model performance was reported using diagnostic characteristics as described for CDA performance. Statistical analysis was performed using IBM statistical package for social sciences (SPSS) version 23 and R statistical software version 4.3.3.

### Cost comparison

A cost comparison was conducted for each CDA (NICE NG143, BSAC, Aronson, AAP) against a treat all approach, as supported by NICE NG51 (Sepsis).^5^ The objectives were to estimate the total health care costs for the cohort under each CDA, along with the mean cost per patient, and the incremental cost (cost saving) of each CDA when compared to the treat all approach. The cost comparison took a NHS (provider) perspective with a short time horizon of 7 days post-initial assessment, in keeping with the cohort study. Health service resource use was collated as part of the CRF and included investigations undertaken, treatments administered, duration of stay in ED (in hours), and, if relevant, duration of hospital stay (in days), reattendance at ED within the time horizon, and ambulatory care (in days). We derived Health Resource Groups (HRGs) at spell level corresponding to the 2022/23 National Tariff to value the health service resource use.^33^ Spell-level HRGs are based on all diagnoses and procedures in the spell. The tariff takes into account type of admission (elective or non-elective) and length of stay. We considered all spells as non-elective and, if length of stay exceeded the trim point for the given HRG, the cost of each excess bed day was added. We sourced the unit cost of PCT testing from guidance on diagnosing and monitoring sepsis,^34^ and uplifted to 2022/2023 prices using the NHS Cost Inflation Index (pay and prices).^35^ Where available, we used Market Forces Factor values for Trust provider to account for unavoidable cost differences based on geographical location.^33^ All unit costs are listed in the Supplementary Table S7. No discount rate was applied due to the time horizon being less than one year. For the cost comparison, a cost profile was presented for each CDA that consisted of ED attendance costs, hospital admission costs, and follow-up health service costs (reattendance to ED and/or ambulatory care). Individual variation in costs was modelled based on the hypothetical performance accuracy of each CDA at correctly identifying infants with and without IBI. Actual patient data were used to formulate the costs for the alternative CDAs, with the assumption that there may be a change in management following classification of febrile infants into lower and higher risk cohorts. Total costs and mean cost per patient were estimated for each CDA based on risk classification of the infant following initial assessment and investigation in ED. Incremental costs (cost savings) were assessed by calculating the mean difference between each CDA and a treat all approach. Bootstrap estimates of the confidence intervals were generated for each point estimate using 10,000 bootstrap replications from the observed data.

### Public and patient involvement (PPI)

PPI in the FIDO study was continuous from commencement, with a specifically convened PPI group including parents of infants with fever presenting to emergency care. The PPI group contributed to study design, including the protocol, study information, and approach to consent.

### Ethics

Ethical approval for the FIDO Study was obtained from the following: the Office for Research Ethics Committees Northern Ireland Health and Social Care Research Ethics Committee B (Ref: 22/NI/0002) and Public Benefit and Privacy Panel for Health and Social Care Scotland (Ref: 2122-0257) and Children's Health Ireland Research and Ethics Committee Ireland (Ref: REC-082-22).

### Study registration

The FIDO study was registered at https://www.clinicaltrials.gov (trial registration: NCT05259683.) on the 28th February 2022.

### Role of the funding source

The funder of the study had no role in study design, data collection, data analysis, data interpretation, or writing of the report. The corresponding author had full access to all the data in the study and had final responsibility for the decision to submit for publication.

## Results

**Fig. 1:**
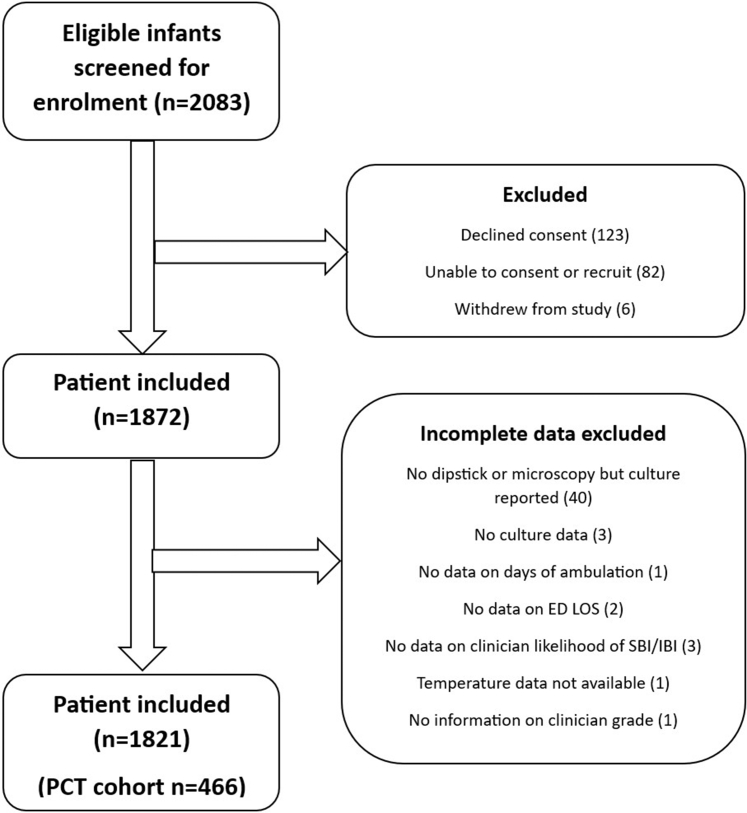
**Flow chart of participant inclusion in the FIDO study**. CDA, Clinical decision aid; ED, Emergency department; FIDO, Febrile Infant Diagnostic Assessment and Outcome; IBI, Invasive bacterial infection; LOS, Length of stay; PCT, Procalcitonin; SBI, Serious bacterial infection.

Between the 6th July 2022 and 31st August 2023 a total of 2083 consecutive infants were screened at 35 sites, of which 211 were excluded (Fig. 1). The remaining 1872 infants were enrolled to the study, 51 (2.7%) of these infants were excluded from analysis due to incomplete CRFs and 1821 were included in the final analysis.

The median age of recruited infants was 46 days (IQR, 30–64) and 1108 (61%) of 1821 infants were male. Comorbidities were reported in 260 of 1821 (14%) of infants and 1058 of 1821 (58%) were deemed to be unwell appearing at presentation (abnormal global assessment n-404 (22%) and abnormal vital sign n-938 (52%)). Most infants underwent diagnostic testing with 1483 of 1821 (81%) undergoing urine testing, 1626 of 1821 (89%) undergoing blood testing, 937 of 1821 (52%) undergoing lumbar puncture, and 1395 of 1821 (77%) undergoing respiratory viral testing. A total of 1242 of 1821 (68%) infants received parenteral antibiotics following initial assessment, 1407 of 1821 (77%) were admitted to hospital with a median length of stay of 2 nights (IQR 2 to 3), 247 of 1821 were discharged (14%), 167 of 1821 (9%) were ambulated, 165 of 1821 (9%) were admitted for observation without parenteral antibiotics, 16 of 1821 (0.9%) were admitted to paediatric intensive care units (PICU) and no infants died. A total of 114 of 1821 (6%) infants reattended emergency care within 7 days of discharge. Of these, 7 were admitted with 2 diagnosed with a UTI. None were subsequently diagnosed with IBI.

In total 67 of the 1821 (3.7%) infants were diagnosed with IBI, including 62 infants with bacteraemia and 9 infants with bacterial meningitis. Of the 67 infants, 4 had concomitant bacteraemia and meningitis. The most reported IBIs were *E. coli* and *Streptococcus agalactiae* accounting for 32 of the 62 (52%) and 10 of 62 (16%) bacteraemia respectively and 1 of 9 (11%) and 3 of 9 (33%) bacterial meningitis respectively. A summary of IBI by type is shown in Table 3. A further 147 of 1821 infants (8%) were diagnosed with UTI of which 114 of 147 (78%) were due to *E. coli* (Table 2).

**Table 3:** Organism isolated in cerebrospinal fluid, blood and urine.

Organism	Total- pathogens N = 218	Meningitis n = 9 (%)	Bacteraemia n = 62 (%)	UTI n = 147 (%)
*Citrobacter freundii*	3	0 (0)	1 (2)	2 (1)
*Enterobacter asburiae*	1	0 (0)	1 (2)	0 (0)
*Enterobacter cloacae*	3	1 (11)	1 (2)	1 (1%)
*Enterococcus faecalis*	5	1 (11)	0 (0)	4 (3)
*Enterococcus* spp.	13	0 (0)	1 (2)	12 (8)
*Escherichia coli*^a^	148	1 (11)	33 (53)	114 (78)
*Klebsiella oxytoca*	2	0 (0)	0 (0)	2 (1)
*Klebsiella pneumoniae*	9	0 (0)	1 (2)	8 (5)
*Neisseria meningitidis*	4	2 (22)	2 (3)	0 (0)
*Proteus mirabilis*	2	0 (0)	0 (0)	2 (1)
*Pseudomonas massiliensis*	1	1 (11)	0 (0)	0 (0)
*Salmonella*	1	0 (0)	1 (2)	0 (0)
*Staphylococcus aureus*	10	0 (0)	9 (15)	1 (1)
*Streptococcus agalactiae (GBS)*	14	3 (33)	10 (16)	1 (1)
*Streptococcus Gallolyticus*	1	0 (0)	1 (2)	0 (0)
*Streptococcus pyogenes*	1	0 (0)	1 (2)	0 (0)

UTI, Urinary tract infection.

a 1 infant had *E. coli and Klebsiella pneumonia* in blood culture. (Culture defined as *E. coli* positive) Of the 67 infants, 4 had concomitant bacteraemia and meningitis, 16 had bacteraemia and UTI. None had concomitant meningitis and UTI.

Univariate analysis of individual clinical features is shown in Table 4. Following multivariable analysis, 4 independent clinical risk factors for IBI were identified: clinician opinion of IBI likely (p < 0.001), no unwell contacts at home (p < 0.001), no rash present (p = 0.044) and absence of cough and coryza (p = 0.005) (Supplementary Table S4). Using the multivariable model derived, positive clinical factors (yes to all including rash/cough&coryza/unwell contacts) along with clinician opinion of IBI unlikely identified 67 infants with no reported IBI. The sensitivity of 1.0 (95% CI 0.95–1.0) and specificity of 0.04 (95% CI 0.03–0.05).”

**Table 4:** Univariate analysis for clinical predictors of IBI (all variables with highlighted p values progressed to multivariable analysis).

Variable	No IBI n-1754 (%)	IBI n-67 (%)	p value	Total with variable reported n (%)
Age median (IQR)	**47 (30–64)**	**31 (16–57)**	**<0.001**	**1821 (100)**
Male sex n (%)	**1056 (60.2)**	**52 (77.6)**	**0.005**	**1821 (100)**
Comorbidities present n (%)	247 (14.1)	13 (19.4)	0.215	1821 (100)
Short duration of fever n (%)	933 (53.2)	33 (49.3)	0.380	1821 (100)
Unwell contacts n (%)	**725 (41.3)**	**9 (13.4)**	**<0.001**	**1821 (100)**
Vaccination within 24 hrs n (%)	**69 (3.9)**	**0 (0)**	**0.178**	**1821 (100)**
Rash n (%)	**335 (19.1)**	**6 (9.0)**	**0.037**	**1821 (100)**
Cough and coryza n (%)	**864 (49.3)**	**17 (25.4)**	**<0.001**	**1821 (100)**
Fever with apparent source n (%)	**1004 (57.2)**	**25 (37.3)**	**0.002**	**1821 (100)**
Meningism present∗ n (%)	**24 (1.4)**	**3 (4.5)**	**0.074**	**1821 (100)**
Temperature median (IQR)	38.0 (37.4–38.4)	38.0 (37.3–38.4)	0.859	1821 (100)
Heart rate median (IQR)	**167 (152–181)**	**173 (159–183)**	**0.132**	**1813 (99.6)**
Respiratory rate median (IQR)	47 (40–52)	46 (40–59)	0.403	1786 (98.1)
Capillary refill time median (IQR)	2 (2–2)	2 (2–3)	0.454	1821 (100)
Unwell appearing n (%)	**1007 (57.4)**	**51 (76.1)**	**0.002**	**1821 (100)**
Oxygen Saturation median (IQR)	**99 (97–100)**	**98 (96–99)**	**<0.001**	**1821 (100)**
Clinician opinion of IBI “likely”	**713 (40.6)**	**55 (82.1)**	**<0.001**	**1821 (100)**

Meningism present if any of the following present, bulging fontanelle, neck stiffness, seizures or focal neurology. Univariate logistic regressions were run using complete case analysis. Bold highlights represent variables that are statistically significant predictor of IBI.

IBI, Invasive bacterial infection; IQR, Inter quartile range.

The BSAC and AAP CDAs demonstrated the highest sensitivities at 0.99 (95% CI 0.92–1.00) for both. Sensitivities of NICE NG143 and the Aronson CDA were 0.93 (95% CI 0.83–0.98) and 0.90 (95% CI 0.80–0.96) respectively. The observed differences in sensitivities between CDAs was not statistically significant (p > 0.05 for all) (Supplementary Table S3). The Aronson and NICE NG143 CDA misclassified 5 and 7 infants with IBI as low risk compared to BSAC and AAP CDA which misclassified 1 infant each (Supplementary Table S5).

The most specific CDAs were NICE NG143 and the Aronson CDAs with a specificity of 0.27 (95% CI 0.25–30) and 0.30 (95% CI 0.28–0.32) respectively. The specificities of NICE NG143 and IBI Score were significantly higher than the specificities of all other CDAs (p < 0.001) (Supplementary Table S3). The BSAC and AAP CDAs performed with specificities of 0.20 (95% CI 0.18–0.22) and 0.23 (95% CI 0.21–0.25) respectively (Table 5). The observed difference in specificity between AAP and BSAC CDAs was significant (p < 0.001).

**Table 5:** Diagnostic performance of NICE, BSAC, Aronson rule and AAP with CRP for IBI in infants ≤90 days.

CDA	Low risk n (%)	IBI in low Risk n (%)	Sensitivity (95% CI)	Specificity (95% CI)	PPV (95% CI)	NPV (95% CI)
NICE NG143	485 (27)	5 (1.0)	0.93 (0.83–0.98)	0.27 (0.25–0.30)	0.05 (0.04–0.06)	0.99 (0.98–1.00)
BSAC	355 (20)	1 (0.3)	0.99 (0.92–1.00)	0.20 (0.18–0.22)	0.05 (0.03–0.06)	1.00 (0.98–1.00)
Aronson rule	526 (29)	7 (1.3)	0.90 (0.80–0.96)	0.30 (0.27–0.32)	0.05 (0.04–0.06)	0.99 (0.97–0.99
AAP^a^	399 (22)	1 (0.3)	0.99 (0.92–1.00)	0.23 (0.21–0.25)	0.05 (0.04–0.06)	1.00 (0.98–1.00)

AAP, American Academy of Pediatrics; BSAC, British Society for Antimicrobial Chemotherapy; CDA, Clinical decision aid; CI, Confidence interval; CRP, C-reactive protein; IBI, invasive bacterial infection; NICE, National Institute for Health and Care Excellence; NPV, Negative predictive value; PPV, Positive predictive value.

a AAP utilising CRP.

Performing the CDA application using either imputation or exclusion of incomplete datasets had no effect on the results (Supplementary Table S2). Excluding infants with co-morbidities (n = 260) made no difference to the performance of the CDAs (Supplementary Table S2). Substituting PCT for CRP (n = 466) made no difference to the performance of the AAP CDA with sensitivities of 0.96 (95% CI 0.80–0.99) and 1.00 (95% CI 0.86–1.00) for PCT and CRP respectively (p = 1.00) and specificities of 0.15 (95% CI 0.12–0.20) and 0.16 (95% CI 0.13–0.20) for PCT and CRP respectively (p = 0.69) (Supplementary Tables S2, S3 and S6).

The total cost for investigation, treatment and follow up under each CDA is presented in Table 6, along with the mean cost per patient and the mean incremental cost savings per patient when compared to a treat all approach. The CDA with the highest mean cost per patient to the NHS was the treat all approach, supported by NICE NG51, at £1537 per patient (bootstrapped 95% CI £1503–£1576), while the Aronson CDA had the lowest mean cost per patient (£1171; bootstrap 95% CI £1129–£1214). When compared to a treat all approach, all of the CDAs produced incremental cost savings that were statistically significant. The more cost-saving strategies were Aronson CDA with mean incremental cost savings per patient of £366 (bootstrap 95% CI £311–£421) and NICE NG143 with mean incremental cost savings per patient of £319 (bootstrap 95% CI £261–£375).

**Table 6:** Cost comparison of each CDA to a treat all approach.

	Total cost per resource category			Total cost per CDA	Mean cost per patient (bootstrap 95% CI)	Mean incremental cost savings per patient (bootstrap 95% CI)
	ED attendance	Hospital admission	Follow-up health services			
Treat all	£806,002	£1,856,706	£135,752	£2,798,459	£1537 (£1503–£1576)	–
NICE NG143	£687,801	£1,405,666	£124,020	£2,217,487	£1218 (1174–£1263)	£319 (£261–£375)
BSAC	£719,318	£1,535,161	£128,828	£2,383,307	£1309 (£1266–£1353)	£228 (£172–£285)
Aronson rule	£677,828	£1,333,692	£120,511	£2,132,031	£1171 (£1129–£1214)	£366 (£311–£421)
AAP–CRP	£708,813	£1,489,690	£128,933	£2,327,495	£1278 (£1236–£1325)	£259 (£202–£316)

AAP, American Academy of Pediatrics; BSAC, British Society for Antimicrobial Chemotherapy; CDA, Clinical decision aid; CI, Confidence interval; CRP, C-reactive protein; ED, Emergency department; IBI, invasive bacterial infection; NICE, National Institute for Health and Care Excellence; NICE NG143, Fever under 5 guideline; Treat all, NICE NG51 (Sepsis guideline).

**Fig. 2:**
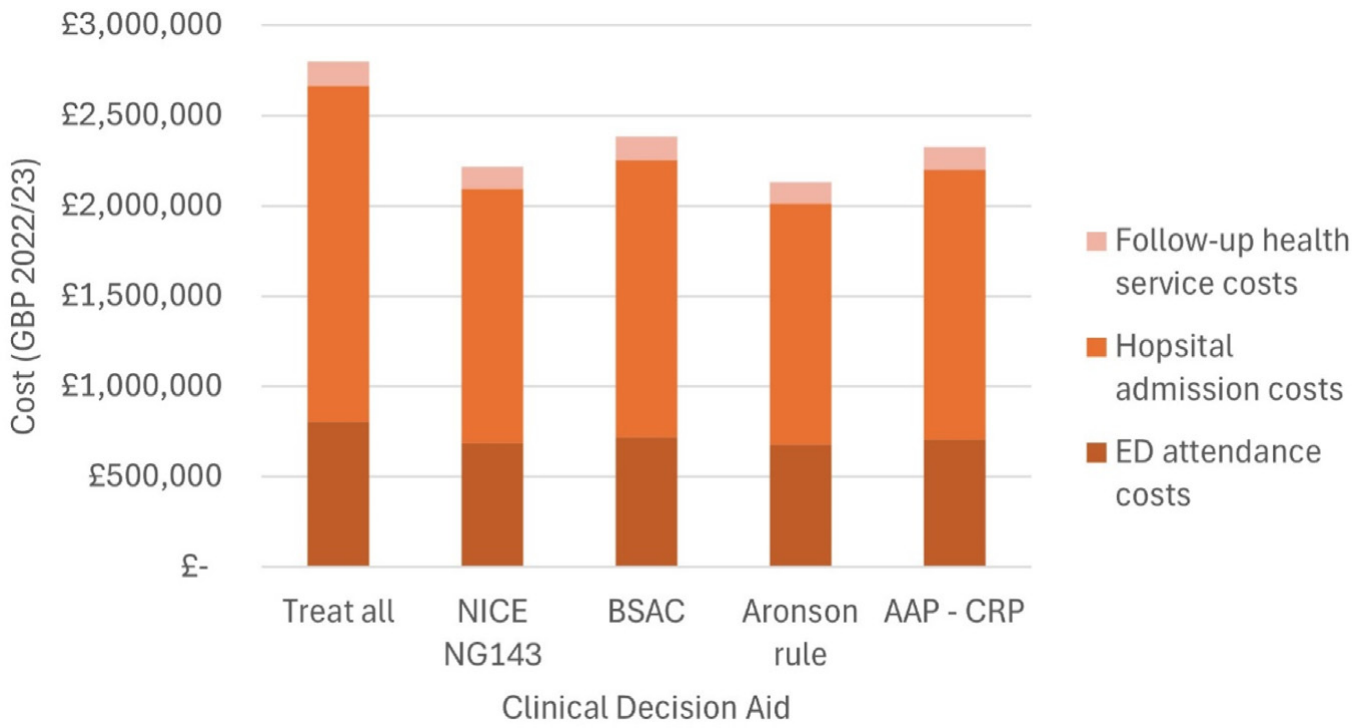
**Cost profile of each CDA versus a treat all approach**. AAP, American Academy of Pediatrics; BSAC, British Society for Antimicrobial Chemotherapy; CDA, Clinical decision aid; CRP, C-reactive protein; ED, Emergency department; NICE, National Institute for Health and Care Excellence; NICE NG143, Fever under 5 guideline; Treat all, NICE NG51 (Sepsis guideline).

The cost profile for each CDA (Fig. 2) illustrated that the driver for savings was the reduced hospital admission costs, due to the change in management and risk stratification following initial assessment and investigation in ED, as well as a reduction in procedures such as lumbar puncture, and fewer infants administered parenteral antibiotics. When the cost of the AAP CDA considered PCT, the mean cost per patient increased from £1278 (bootstrap 95% CI £1236–£1325) to £1396 (bootstrap 95% CI £1318–£1479) (Supplementary Table S2).

## Discussion

The FIDO study represents the largest study of young febrile infants performed in the UK and Ireland to date. Infants were enrolled from a diverse range of hospital types and geographic regions over 12 months to provide an accurate description of epidemiology, clinician practices, and performance of CDAs.

The proportion of infants with an IBI was 3.7% in our cohort, in keeping with similar studies conducted across Europe and North America.^2^^,^^3^^,^^20^ Most cases of IBI in our study were due to either *E. coli* or *Streptococcus agalactiae*, typically causing bacteriaemia or bacterial meningitis respectively. These findings are consistent with other published literature which demonstrates that over the last two decades, IBI epidemiology has changed. Bacteria such as *Streptococcus pneumoniae*, *Haemophilus influenza*e and *Listeria monocytogenes* have become less frequent, and *E. coli* has emerged as the most frequent cause.^2^^,^^4^^,^^36^ This observed shift has largely been attributed to changes in antenatal care, vaccinations and public health measures.^6^^,^^9^^,^^37^^,^^38^

Of the assessed CDAs, the AAP and BSAC were the most sensitive (99%) with only 1 lower risk infant each subsequently diagnosed with IBI. Both infants were aged between 29 and 60 days and presented within 6 h of fever onset. Infants with a short duration of fever onset require careful consideration and may benefit from a period of observation. Whilst highly sensitive, both the AAP and BSAC CDAs identified a clinically significant lower risk cohort of 20% (BSAC) and 22% (AAP) that could be managed without lumbar puncture and immediate parenteral antibiotics. Substituting CRP for PCT in the AAP CDA made no difference to the diagnostic performance but increased the mean cost per infant from £1278 to £1396. The Aronson rule and NICE CG143 were the most specific CDAs, identifying lower risk cohorts of 29% and 27% respectively. However, they were poorly sensitive with 5 and 7 infants with IBI deemed as lower risk by NICE NG51 and the IBI score respectively. For the NICE NG143 CDA, misclassified infants had elevated CRP, while for the Aronson CDA, misclassified infants were more likely to appear unwell. Both NICE NG143 and Aronson CDAs use WCC and ANC as biomarkers, neither incorporating CRP or PCT. Studies have shown that WCC and ANC are inferior to CRP and PCT in terms of diagnostic performance for febrile infants.^39^^,^^40^ The performance of the Aronson rule was similar to previously published validation studies.^17^^,^^23^ Both the Aronson and NICE NG143 had the lowest mean cost per patient reported as £1171 (bootstrap 95% CI £1129–£1214) and £1218 (bootstrap 95% CI £1174–£1263) when compared to a treat all approach. This was due to their higher specificity and ability to identify a low risk cohort. Our results can help inform decision making by policy makers when deciding on which CDA to implement.

All of the CDAs utilised a combination of clinical features and biomarker predictors of IBI with age and clinical appearance common to most. The multivariable analysis identified 4 independent clinical predictors of IBI including absence of cough and coryza, no reported rash, no unwell contact at home and clinician opinion of IBI being likely. Clinician opinion of IBI being likely identified 82% of infants with IBI. This could reflect clinician experience and use of known clinical risk factors in the assessment of febrile infants. When this combination of clinical factors were present (presence of rash, unwell contact, respiratory symptoms (cough/coryza) and clinician opinion of IBI being unlikely), the model identified 4% of infants that could be managed without need for phlebotomy. These clinical factors could be used a clinical screen in terms of management of febrile infants prior to phlebotomy.

Overall, 9% of the study population were admitted for observation without immediate parenteral antibiotics. The ability to observe, over time, for signs of deterioration may have influenced clinician confidence with sequential assessment and raises the issue of how to manage lower risk infants. Admitting well appearing lower risk infants for a period of observation may represent a reasonable compromise that could reduce overall length of stay and improve antimicrobial stewardship. An alternative to admission could be ambulation and could lead to better cost savings if implemented. If adopting such a practice both low risk and some high-risk patient could be ambulated and represent a paradigm shift in the management of febrile infants. The AAP and recent published Canadian febrile infant (>28 days of age) guidelines advocate for discharge home of low risk infants with a negative urinalysis with no antibiotics and arrange follow up in 24–48 h.^6^^,^^41^ However, they also give the option to admit and observe for 24 h for clinical observation until all cultures are negative within 24 h.^6^^,^^41^ Shared decision marking and communication of risk is paramount to enabling effective implementation of these strategies.^42^^,^^43^

The FIDO study has many strengths including the numbers of infants included, the prospective recruitment and the geographical spread of included sites. The main limitations are that only a small proportion of the total cohort underwent PCT testing. Whilst no difference in CDA performance was found on sensitivity analysis, the study was underpowered to detect very small differences in CDA performance. It remains, however, unclear if such small differences would be clinically relevant. In addition, not all infants underwent biomarker testing as standard clinical practice was followed. Exclusion of these infants in the sensitivity analysis did not affect CDA performance. Another limitation is that infants were followed up through hospital chat review for reattendance and eventual IBI diagnosis. This pragmatic approach is typical of similar cohort studies but this methodology does introduce a small risk of underestimating IBI rates.^44^^,^^45^ With this in mind, it is reassuring that the observed IBI rate in this study was 3.7% similar to international studies of this nature.^2^, ^3^, ^4^

In this large multicentre study evaluating the management of febrile infants across the UK and Ireland, the rate of IBI was 3.7%, with *E. coli* being the most common cause. The AAP and BSAC CDAs showed the highest sensitivity, while the Aronson rule and NICE NG143 were most cost-effective due to higher specificity. Substituting CRP for PCT in the AAP CDA made no significant difference in diagnostic performance. Our study suggests a more nuanced approach to managing febrile infants, potentially including observation periods for lower-risk infants and ambulation strategies.

## Contributors

EU, CM, HNB, HM, LM, FL, DR, MDL, CW, and TW were involved in the conception and design of this study. EU, CM, SF, MJB and TW coordinated the running of the study including data management and site training. EU, CM, GM, CW, and TW designed sample collection, storage, and analysis. EU, FL, and TW designed and undertook for the economic analysis evaluation. EU, TW, HM and LM have approved and verified the underlying data. EU, HM, and LM undertook the statistical analysis. All authors reviewed and approved the final manuscript.

## Data sharing statement

Data Sharing All of the individual participant data collected during this study will be available (including data dictionaries) on the Queen's University Belfast data repository. The full study protocol is available as an open access publication.

## Declaration of interests

No conflict of interest to declare.

## References

[1] Ladhani S.N., Henderson K.L., Muller-Pebody B., Ramsay M.E., Riordan A. (2019). Risk of invasive bacterial infections by week of age in infants: prospective national surveillance, England, 2010–2017. Arch Dis Child.

[2] Kuppermann N., Dayan P.S., Levine D.A. (2019). A Clinical prediction rule to identify febrile infants 60 days and younger at low risk for serious bacterial infections. JAMA Pediatr.

[3] Gomez B., Mintegi S., Bressan S., Da Dalt L., Gervaix A., Lacroix L. (2016). Validation of the ‘step-by-step’ approach in the management of young febrile infants. Pediatrics.

[4] Waterfield T., Lyttle M.D., Munday C. (2021). Validating clinical practice guidelines for the management of febrile infants in the United Kingdom and Ireland. Arch Dis Child.

[5] National Institute for Health and Care Excellence (2020).

[6] Pantell R.H., Roberts K.B., Adams W.G. (2021). Clinical practice guideline: evaluation and management of Well-Appearing febrile infants 8 to 60 days old. Pediatrics.

[7] Leazer R., Perkins A.M., Shomaker K., Fine B. (2016). A meta-analysis of the rates of Listeria monocytogenes and Enterococcus in febrile infants. Hosp Pediatr.

[8] Woll C., Neuman M., Pruitt C. (2018). Epidemiology and etiology of invasive bacterial infection in infants <= 60 days old treated in emergency departments. J Pediatr.

[9] Greenhow T.L., Hung Y.Y., Herz A.M., Losada E., Pantell R.H. (2014). The changing epidemiology of serious bacterial infections in young infants. Pediatr Infect Dis J.

[10] Watt K., Waddle E., Jhaveri R. (2010). Changing epidemiology of serious bacterial infections in febrile infants without localizing signs. PLoS One.

[11] Biondi E., Evans R., Mischler M. (2013). Epidemiology of bacteremia in febrile infants in the United States. Pediatrics.

[12] Gomez B., Fernandez-Uria A., Benito J., Lejarzegi A., Mintegi S. (2021). Impact of the Step-by-Step on febrile infants. Arch Dis Child.

[13] Foster L.Z., Beiner J., Duh-Leong C. (2020). Implementation of febrile infant management guidelines reduces hospitalization. Pediatr Qual Saf.

[14] National Institute for Health and Care Excellence (2019).

[15] Sanjay P., Robert Cunney A., Demirjian (2020). https://bsac.org.uk/.

[16] Aronson P.L., Shabanova V., Shapiro E.D. (2019). A prediction model to identify febrile infants≤ 60 days at low risk of invasive bacterial infection. Pediatrics.

[17] Tsai S.J., Ramgopal S. (2021). External validation of an invasive bacterial infection score for young febrile infants. Hosp Pediatr.

[18] Sutiman N., Khoo Z.X., Ong G.Y.K., Piragasam R., Chong S.L. (2022). Validation and comparison of the PECARN rule, Step-by-Step approach and Lab-score for predicting serious and invasive bacterial infections in young febrile infants. Ann Acad Med Singapore.

[19] Velasco R., Gomez B., Benito J., Mintegi S. (2021). Accuracy of PECARN rule for predicting serious bacterial infection in infants with fever without a source. Arch Dis Child.

[20] Nguyen T.H.P., Young B.R., Alabaster A. (2023). Using AAP guidelines for managing febrile infants without C-reactive protein and procalcitonin. Pediatrics.

[21] Umana E., Mills C., Norman-Bruce H. (2023). Applying clinical decision aids for the assessment and management of febrile infants presenting to emergency care in the UK and Ireland: febrile Infant Diagnostic Assessment and Outcome (FIDO) Study protocol. BMJ Open.

[22] Burstein B., Alathari N., Papenburg J. (2022). Guideline-based risk stratification for febrile young infants without procalcitonin measurement. Pediatrics.

[23] Ramgopal S., Horvat C.M., Yanamala N., Alpern E.R. (2020). Machine learning to predict serious bacterial infections in young febrile infants. Pediatrics.

[24] Norman-Bruce H., Umana E., Mills C. (2024). Diagnostic test accuracy of procalcitonin and C-reactive protein for predicting invasive and serious bacterial infections in young febrile infants: a systematic review and meta-analysis. Lancet Child Adolesc Health.

[25] Collins G.S., Reitsma J.B., Altman D.G., Moons K.G.M. (2015). Transparent reporting of a multivariable prediction model for individual prognosis or diagnosis (TRIPOD): the TRIPOD Statement. BMC Med.

[26] Bossuyt P.M., Reitsma J.B., Bruns D.E. (2015). Stard 2015: an updated list of essential items for reporting diagnostic accuracy studies. BMJ.

[27] Husereau D., Drummond M., Augustovski F. (2022). Consolidated Health Economic Evaluation Reporting Standards 2022 (CHEERS 2022) statement: updated reporting guidance for health economic evaluations. BMC Med.

[28] Wilcox H., Umana E., Fauteux-Lamarre E., Velasco R., Waterfield T. (2024). Conundrums in the management of febrile infants under three months of age and future research. Antibiotics.

[29] Harris P.A., Taylor R., Thielke R., Payne J., Gonzalez N., Conde J.G. (2009). Research electronic data capture (REDCap)-A metadata-driven methodology and workflow process for providing translational research informatics support. J Biomed Inf.

[30] Harris P.A., Taylor R., Minor B.L. (2019). The REDCap consortium: building an international community of software platform partners. J Biomed Inf.

[31] Azur M.J., Stuart E.A., Frangakis C., Leaf P.J. (2011). Multiple imputation by chained equations: what is it and how does it work?. Int J Methods Psychiatr Res.

[32] Smith S. (2023).

[33] 2022-23 National Tariff Payment System (2022). NHS England and NHS Improvement. https://www.england.nhs.uk/wp-content/uploads/2020/11/22-23-National-tariff-payment-system.pdf.

[34] Procalcitonin testing for diagnosing and monitoring sepsis (ADVIA Centaur BRAHMS PCT assay, BRAHMS PCT Sensitive Kryptor assay, Elecsys BRAHMS PCT assay, LIAISON BRAHMS PCT assay and VIDAS BRAHMS PCT assay).

[35] Jones K.C., Weatherly H., Birch S. (2023). https://www.pssru.ac.uk/unitcostsreport/.

[36] Bressan S., Gomez B., Mintegi S. (2012). Diagnostic performance of the Lab-score in predicting severe and invasive bacterial infections in well-appearing young febrile infants. Pediatr Infect Dis J.

[37] Poehling K.A., Talbot T.R., Griffin M.R. (2006 Apr 12). Invasive pneumococcal disease among infants before and after introduction of pneumococcal conjugate vaccine. JAMA.

[38] Ohlsson A., Shah V.S. (2014). Intrapartum antibiotics for known maternal Group B streptococcal colonization. Cochrane Database Syst Rev.

[39] Gomez B., Bressan S., Mintegi S. (2012). Diagnostic value of procalcitonin in well-appearing young febrile infants. Pediatrics.

[40] Milcent K., Faesch S., Guen C.G.L. (2016). Use of procalcitonin assays to predict serious bacterial infection in young febrile infants. JAMA Pediatr.

[41] Burstein B., Lirette M.P., Beck C., Chauvin-Kimoff L., Chan K. (2024). Management of well-appearing febrile young infants aged ≤90 days. Paediatr Child Health.

[42] Aronson P.L., Schaeffer P., Niccolai L.M., Shapiro E.D., Fraenkel L. (2021). Parents' perspectives on communication and shared decision making for febrile infants ≤60 Days old. Pediatr Emerg Care.

[43] Wilson K., Umana E., McCleary D., Waterfield T., Woolfall K. (2024). Exploring communication preferences and risk thresholds of clinicians and parents of febrile infants under 90 days presenting to the emergency department: a qualitative study. Arch Dis Child.

[44] Orfanos I., Elfving K., Sotoca Fernandez J. (2022). Management and outcome of febrile infants ≤60 days, with emphasis on infants ≤21 Days old, in Swedish pediatric emergency departments. Pediatr Infect Dis J.

[45] McCulloh R.J., McDaniel L.M., Kerns E., Biondi E.A. (2021). Prevalence of invasive bacterial infections in well-appearing, febrile infants. Hosp Pediatr.

