# Supplementary material for: Performance of clinical decision aids (CDA) for the care of young febrile infants: a multicentre prospective cohort study conducted in the UK and Ireland
Source: eClinicalMedicine. 2024 Nov 27;78:102961. doi: 10.1016/j.eclinm.2024.102961 (PMC11638619; doi:10.1016/j.eclinm.2024.102961)
Supplement: Supplementary Tables [file mmc1.docx]

**Supplementary Material (Fig S1 – S2, Tables S1 – S9, Study definition and CDA application, Procalcitonin analysis)**

Table of Contents

[Figure S1 :1 Case report form 1. 2](#_Toc181700251)

[Figure S2: Case report form 2. 4](#_Toc181700252)

[Figure S3: Adjudication guidance for classification of bacterial infection (Supplied by Dr Roberto Velasco) 7](#_Toc181700253)

[Study definitions and CDA application 8](#_Toc181700254)

[Procalcitonin (PCT) analysis 8](#_Toc181700255)

[Table S1: Site characteristics and recruitment 9](#_Toc181700256)

[Table S2: Sensitivity analysis for the diagnostic performance of NICE, BSAC, Aronson rule and AAP with CRP or PCT for IBI in infants ≤ 90 days with main analysis, imputed data and exclusions. 10](#_Toc181700257)

[Table S3: Mcnemar test with Bonferroni correction for CDA main analysis, imputation and with exclusions. 11](#_Toc181700258)

[Table S4: Stepwise binary logistic regression to predict IBI 13](#_Toc181700259)

[Table S5. Infants with IBI misclassified by the CDAs (NICE, BSAC, Aronson and AAP-CRP) 14](#_Toc181700260)

[Table S6: Infant misclassified by AAP-PCT CDA in cohort with PCT available (n-466) 15](#_Toc181700261)

[Table S7: Unit costs and sources 16](#_Toc181700262)

[Table S8: Participating sites and investigators (Paediatric Emergency Research in the UK and Ireland (PERUKI) 17](#_Toc181700263)

## Figure S1 :1 Case report form 1.


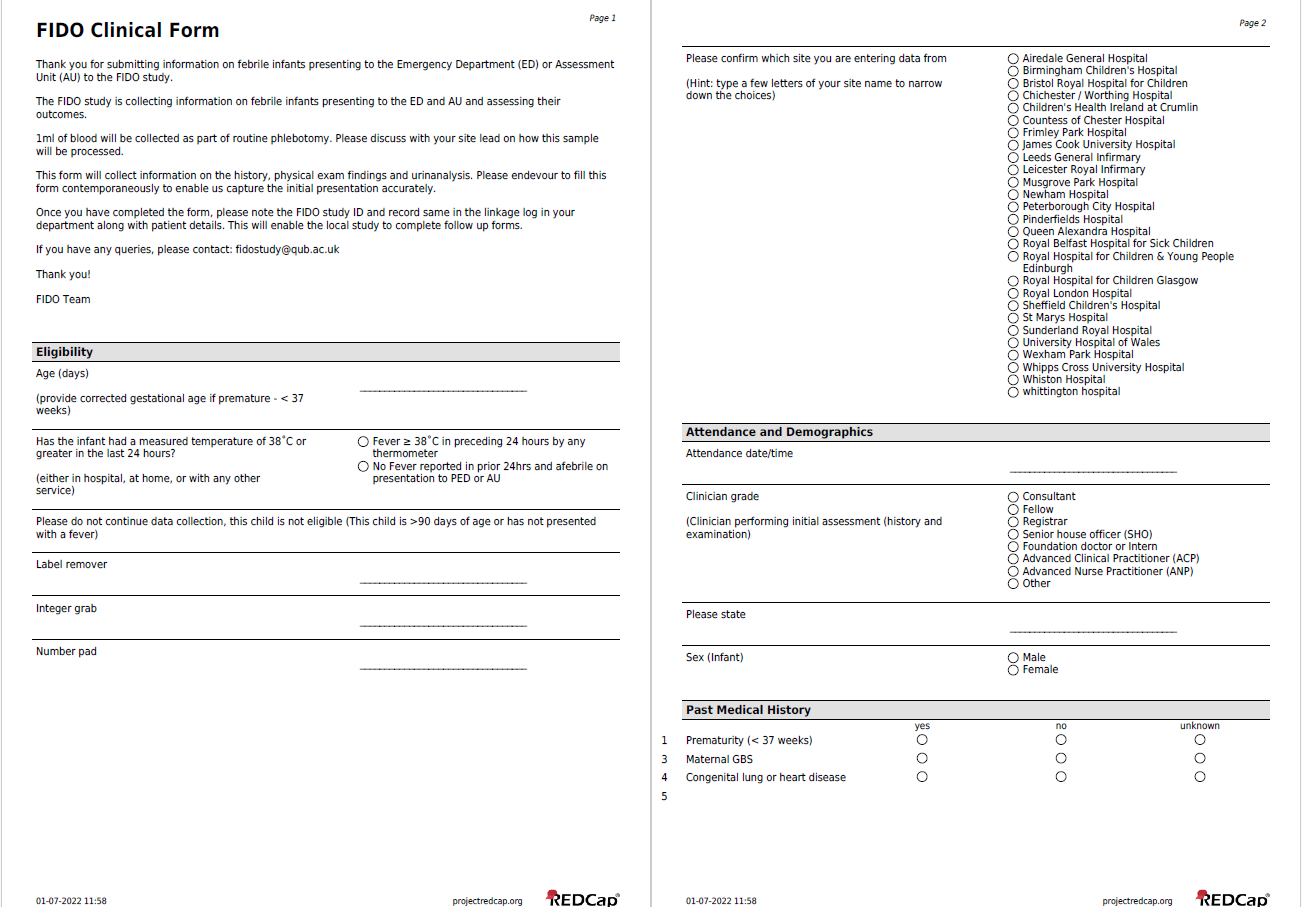


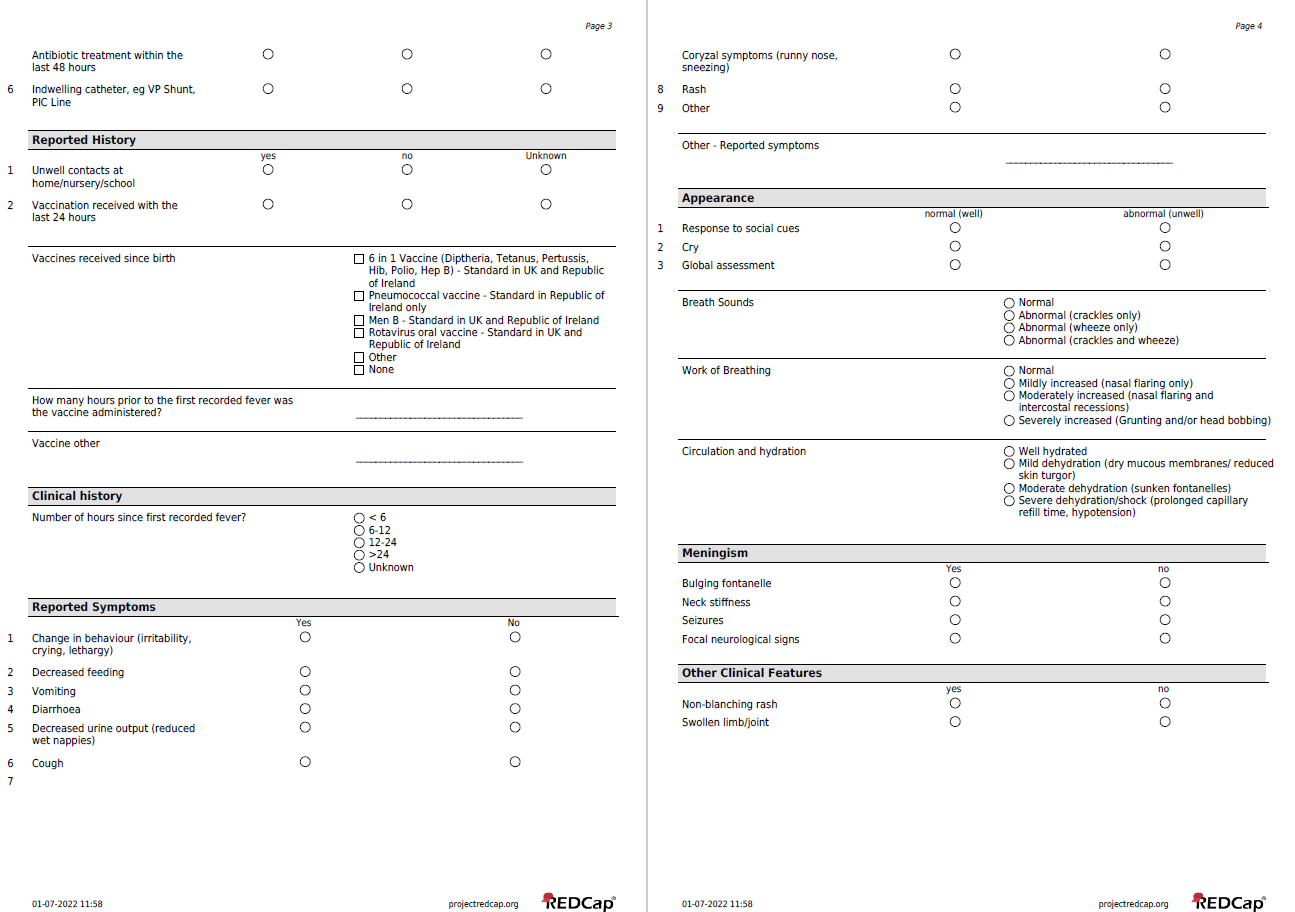


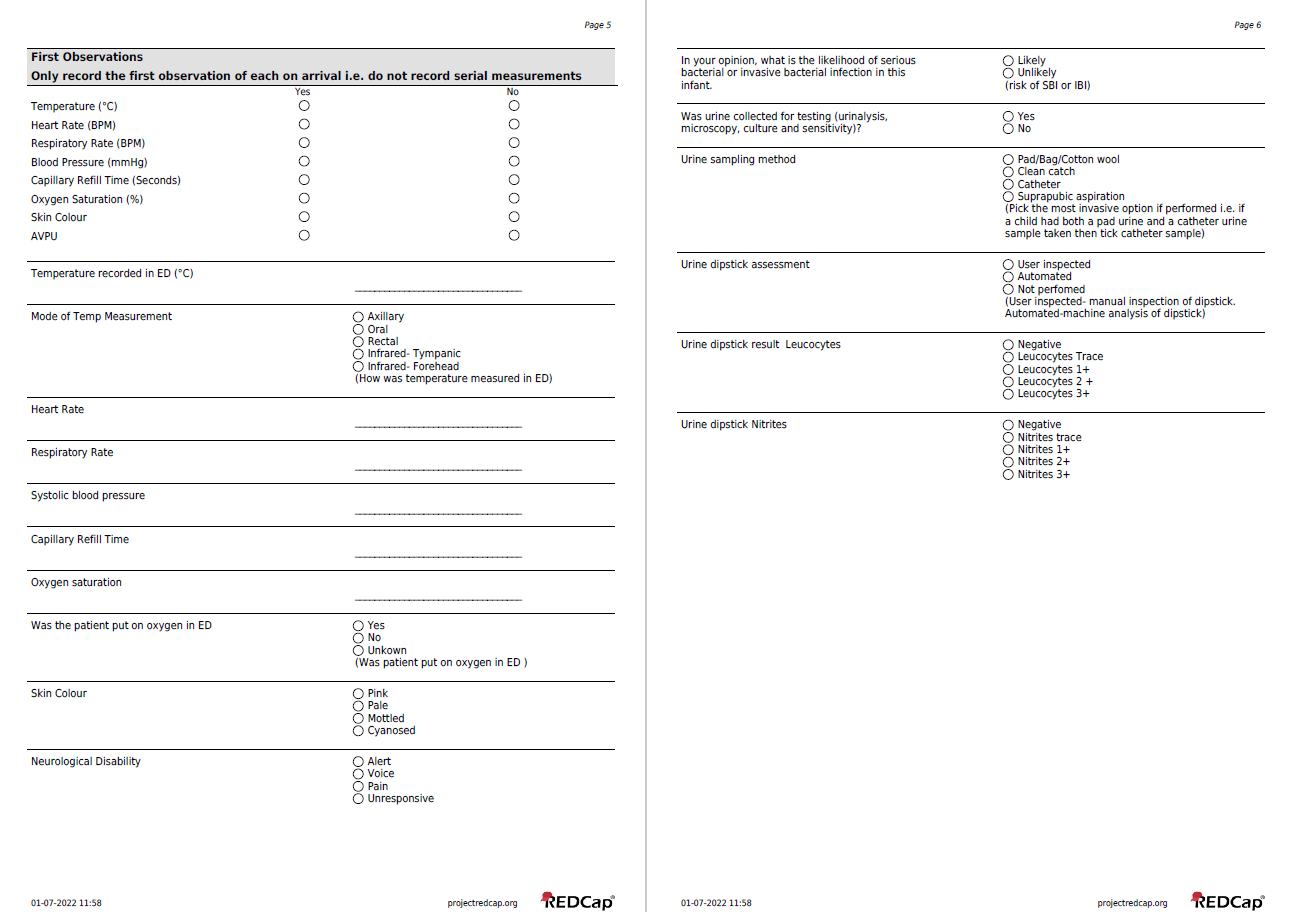


## Figure S2: Case report form 2.


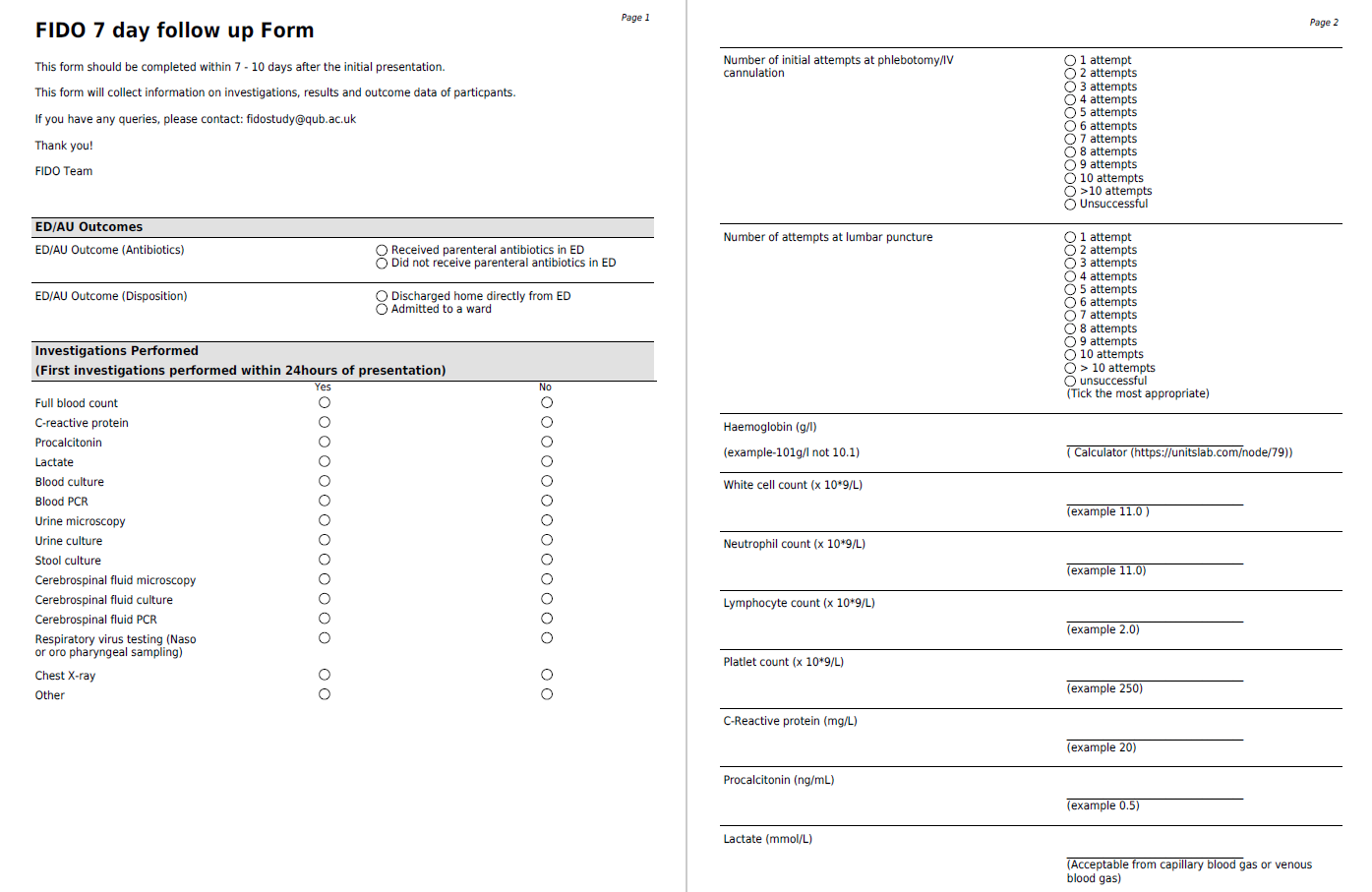


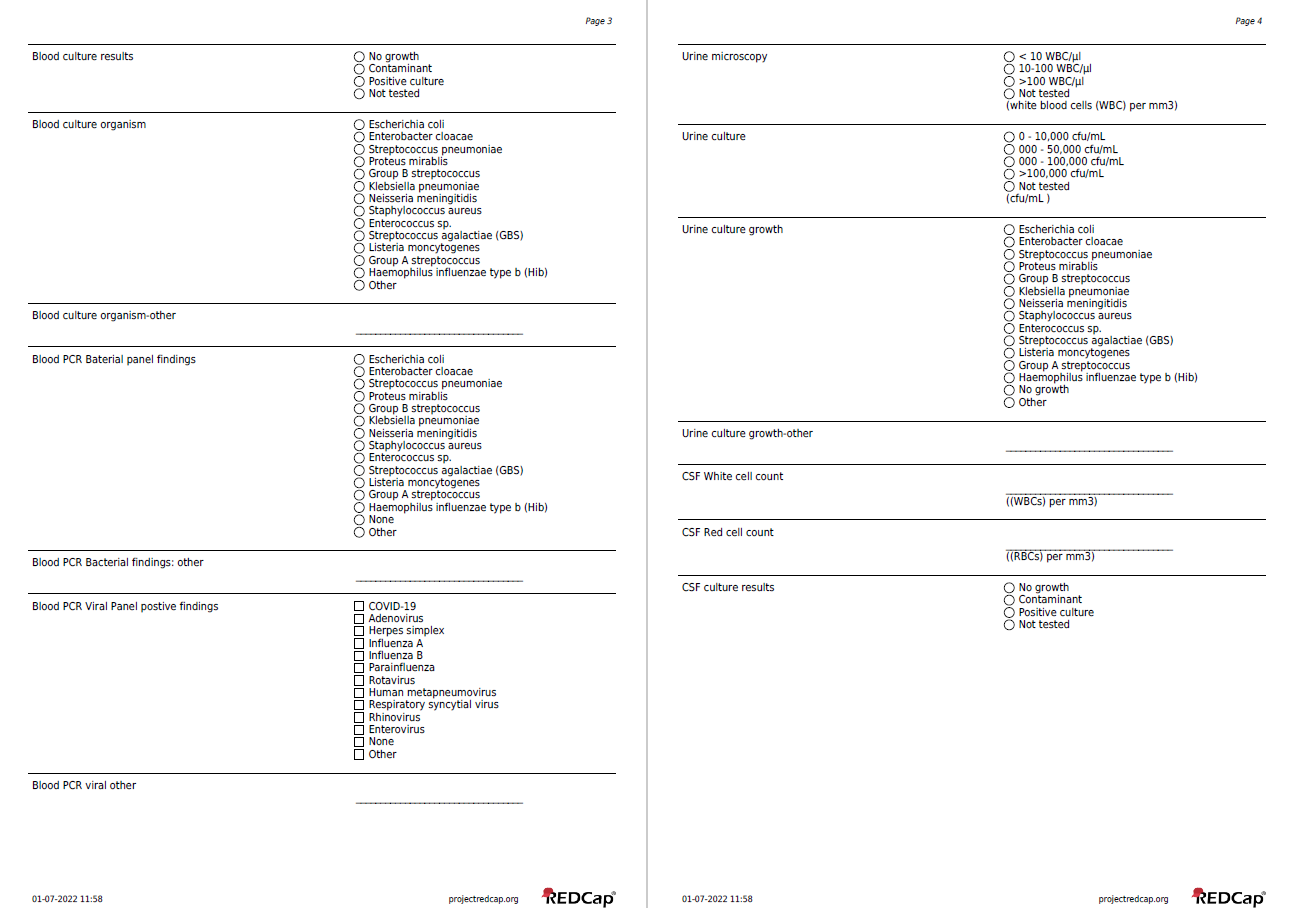


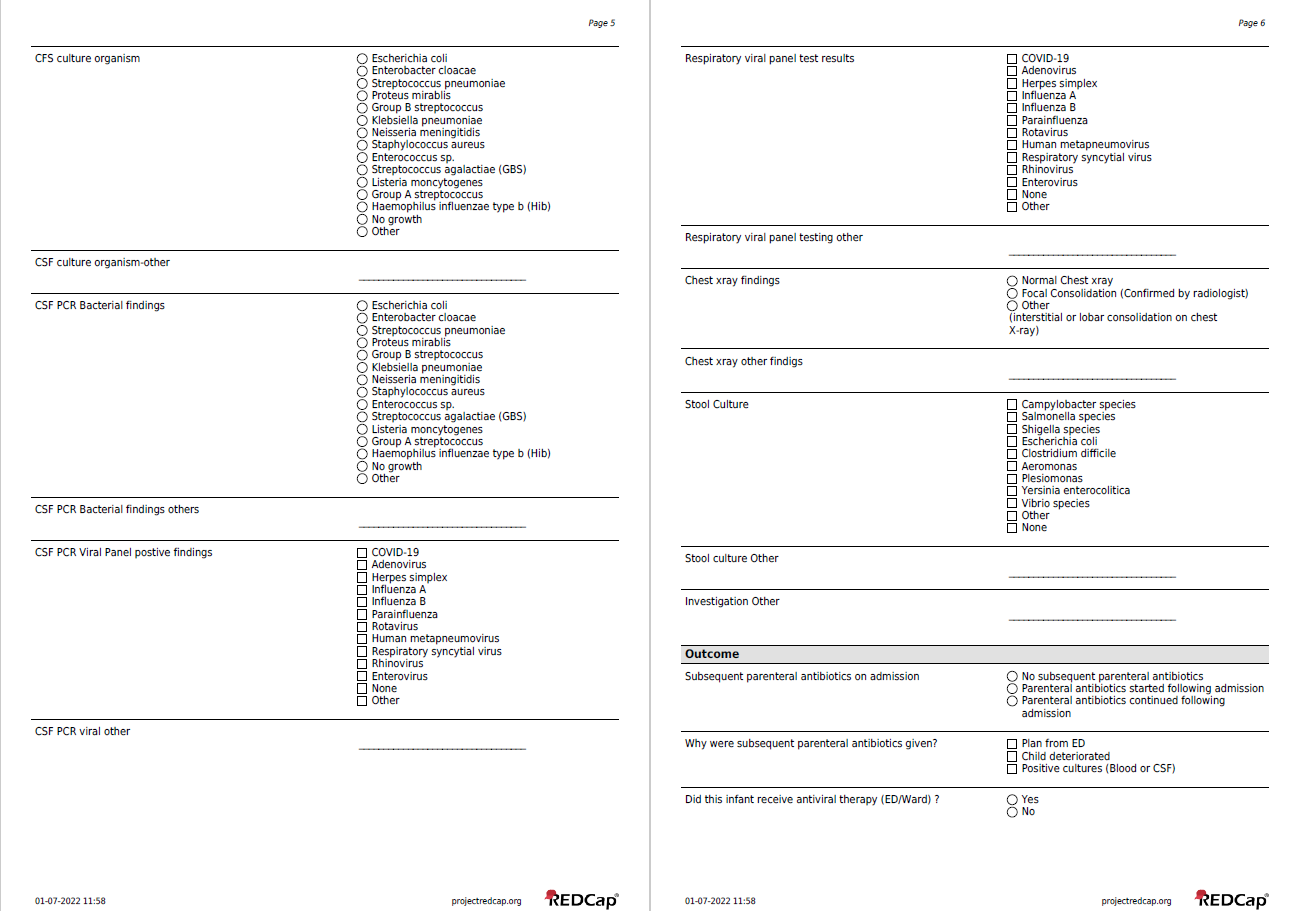


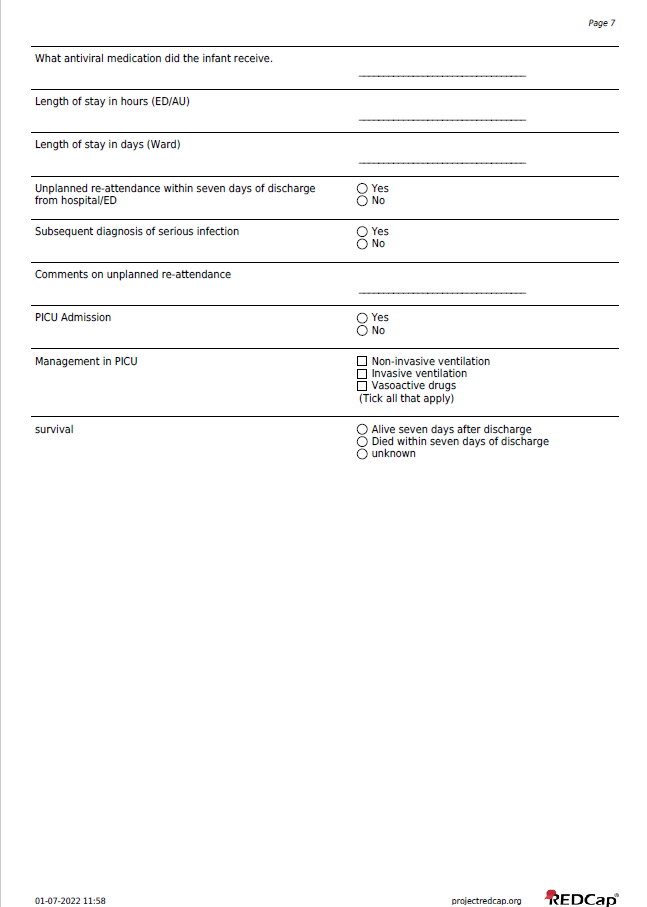


## Figure S3: Adjudication guidance for classification of bacterial infection (Supplied by Dr Roberto Velasco)


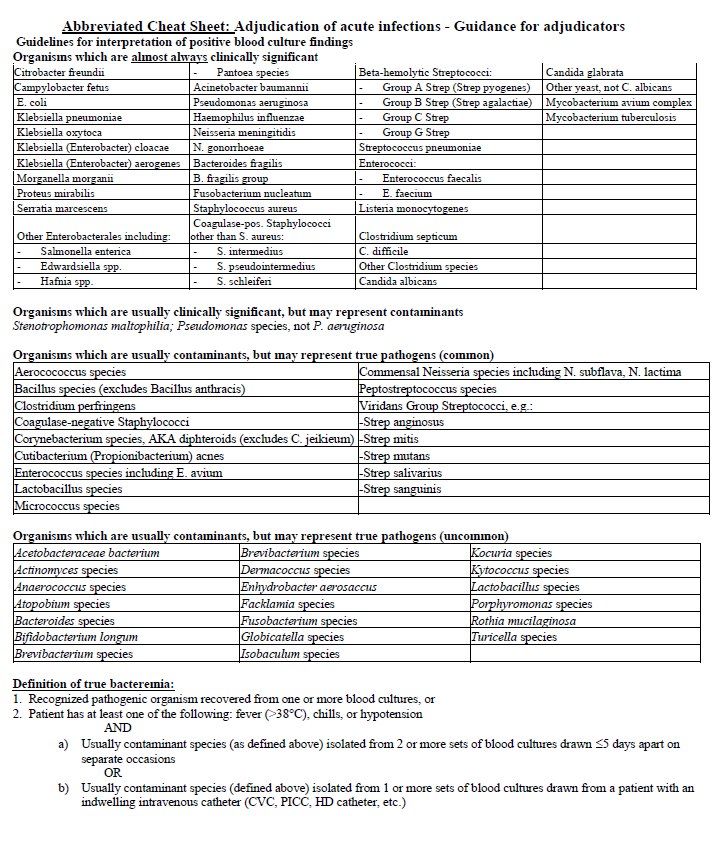


## Study definitions and CDA application

**Urinary tract infection (UTI**) (3,4). The presence of a UTI was confirmed by either:

• Pure growth greater than 100 000 CFU/mL of a single organism from a single clean catch urine sample.

• Pure growth greater than 10 000 CFU/mL of single organism from either a trans-urethral bladder catheter (TUBC) or suprapubic aspiration (SPA) urine sample.

• Pure growth, from two urine samples, greater than 100 000 CFU/mL of the same organism from urine pad/bag samples and pyuria (>10 white blood cells per high-power field) on laboratory microscopy

**Urinalysis criteria** (4): Urinalysis criteria: (1) abnormal urinary dipstick test (leucocyte esterase ≥1+, or nitrite ≥Trace) or (2) abnormal urine microscopy (≥ 10 white cells per high-power field in centrifuged urine) (3) where urinalysis was not obtained and no UTI within 7 day follow up post discharge, was considered to be normal.

**Unwell appearing** (32): Infant were considered unwell if they had an abnormal global assessment and or abnormal vital signs (based on APLS recommended for age).

**Fever with a source (FWS)** (3): defined as any infant after careful history and exam a likely source of fever is identified.

**Application of CDAs** (4,6,15,16): This was based on a stepwise approach on low risk criteria for each. Briefly, low risk criteria for NICE NG143 are >1 month, well appearing, WCC 5-10 X 10^9/L. The BSAC CDA low risk criteria are >1 month and well appearing, negative urinalysis and CRP <20mg/L. This was recently updated after 2021 to include ANC <10 X 10^9/L. For the IBI score, cutoff of 2 is considered low risk. The score ranges from 0 - 10 according to the following points: newborn < 21 days old: 1 point, body temperature 38.0-38.4 °C: 2 points, body temperature > 38.4 °C: 4 points, abnormal urinalysis result: 3 points, ANC >5.2 X 10^9/L: 2 points. For the AAP CDA with CRP the following was considered low risk, >21 days, appearing well, fever <38.5C, ANC <5.2 X 10^9/L and CRP <20mg/L. For the AAP CDA with PCT the following low risk criteria was used, >21 days, appearing well, ANC <4 X 10^9/L and PCT <0.5ng/mL. As PCT was not available for all infants, analysis for AAP with PCT was only conducted in those patients with PCT available and analysis of AAP CDA with CRP repeated for that subgroup.

## Procalcitonin (PCT) analysis

PCT measurements was performed on the Roche Cobas® 702 analyser. PCT was measured by electrochemiluminescence immunoassay (ECLIA), using the Elecsys BRAHMS PCT assay. This immunoassay uses the sandwich principle whereby a biotinylated monoclonal

PCT‑specific antibody and a monoclonal PCT‑specific antibody labelled with a ruthenium complex) react with antigen in the sample to form a sandwich complex. This complex is subsequently bound to a solid phase via interaction of biotin and streptavidin, and magnetically captured in a measuring cell before unbound substances are removed. The application of voltage induces a chemiluminescent emission which is measured by a photomultiplier and results are determined via a calibration curve. This method has been standardized against the BRAHMS PCT LIA assay and has a measuring range of 0.02 – 100 ng/mL with an intermediate precision in human serum of 5% and 1.5% at concentrations of 0.09 and 97.8 ng/mL, respectively

## Table S1: Site characteristics and recruitment

| **Sites/trust names** | **Numbers of months open** | **Cat Annual census** | **Site population** | **Recruited patients** | **Guidelines** |
| --- | --- | --- | --- | --- | --- |
| Airedale Hospital (AIR) | 13 | < 20000 | Mixed (Adults and Peadiatric) | 28 | NICE NG51 |
| Alder Hey Children's Hospital NHS Foundation Trust, Liverpool (ALD) | 5 | > 40000 | Peadiatric only | 78 | NICE NG143 |
| Bradford Royal Infirmary (BRD) | 7 | 20000 - 40000 | Mixed (Adults and Peadiatric) | 11 | NICE NG51 |
| Bristol Royal Hospital for Children (BRI) | 13 | > 40000 | Peadiatric only | 105 | NICE NG143 |
| Children's Health Ireland at Crumlin (CHI) | 4 | > 40000 | Peadiatric only | 59 | None |
| Countess of Chester NHS Foundation Trust (CHS) | 13 | < 20000 | Mixed (Adults and Peadiatric) | 30 | NICE NG143 |
| Denmark Hill (Kings College NHSFT) (KCH) | 3 | > 40000 | Mixed (Adults and Peadiatric) | 8 | NICE NG51 |
| James Cook University Hospital, Middlesbrough (MID) | 11 | 20000 - 40000 | Mixed (Adults and Peadiatric) | 55 | NICE NG51 |
| Leeds General Infirmary (LGI) | 7 | 20000 - 40000 | Peadiatric only | 65 | NICE NG143 |
| Leicester Royal Infirmary (LRI) | 9 | > 40000 | Peadiatric only | 84 | NICE NG51 |
| Musgrove Park Hospital, Taunton (MUS) | 11 | < 20000 | Mixed (Adults and Peadiatric) | 26 | NICE NG51 |
| Newham Hospital, London (NWH) | 12 | > 40000 | Peadiatric only | 44 | NICE NG143 |
| North Tees Hospital (NTH) | 11 | < 20000 | Mixed (Adults and Peadiatric) | 29 | NICE NG143 |
| Peterborough City Hospital (PET) | 8 | < 20000 | Mixed (Adults and Peadiatric) | 39 | NICE NG51 |
| Pinderfield Hospital (PND) | 8 | > 40000 | Peadiatric only | 17 | NICE NG143 |
| Poole Hospital (POH) | 10 | 20000 - 40000 | Mixed (Adults and Peadiatric) | 86 | NICE NG143 |
| Queen Alexandra Hospital, Portsmoouth (QAH) | 12 | 20000 - 40000 | Peadiatric only | 73 | NICE NG51 |
| Royal Alexandra Children's Hospital, Brighton (RAC) | 8 | 20000 - 40000 | Peadiatric only | 46 | NICE NG143 |
| Royal Belfast Hospital for Sick Children (BEL) | 11 | > 40000 | Peadiatric only | 85 | BSAC |
| Royal Berkshire NHS Foundation Trust (BER) | 7 | > 40000 | Peadiatric only | 54 | None |
| Royal Cornwall Hospital NHSFT (CNH) | 7 | 20000 - 40000 | Peadiatric only | 18 | NICE NG143 |
| Royal Hospital for Children & Young People, Edinburgh (EDI) | 6 | > 40000 | Peadiatric only | 102 | None |
| Royal Hospital for Children, Glasgow (GLA) | 7 | > 40000 | Peadiatric only | 90 | None |
| Sheffield Children's NHS Foundation Trust (SCH) | 8 | > 40000 | Peadiatric only | 64 | NICE NG143 |
| South Tyneside & Sunderland NHS Foundation Trust (SUN) | 11 | 20000 - 40000 | Peadiatric only | 86 | NICE NG51 |
| Southampton Children's Hospital (UHS) | 6 | 20000 - 40000 | Peadiatric only | 50 | NICE NG143 |
| St Helen's & Knowsley NHS Trust (SHK) | 13 | 20000 - 40000 | Mixed (Adults and Peadiatric) | 17 | NICE NG51 |
| St Mary's Hospital (MAR) | 9 | 20000 - 40000 | Mixed (Adults and Peadiatric) | 50 | NICE NG143 |
| St Richard's Hospital, Chichester (STR) | 9 | 20000 - 40000 | Mixed (Adults and Peadiatric) | 30 | NICE NG143 |
| The Royal London Hospital (BAR) | 12 | > 40000 | Mixed (Adults and Peadiatric) | 87 | NICE NG143 |
| University Hospital of Wales, Cardiff (CAR) | 9 | 20000 - 40000 | Peadiatric only | 42 | NICE NG143 |
| Watford General Hospital (WGH) | 8 | 20000 - 40000 | Peadiatric only | 18 | NICE NG51 |
| Whipps Cross Hospital, London (WXH) | 12 | 20000 - 40000 | Peadiatric only | 31 | NICE NG143 |
| Whittington Health NHS Trust (WHI) | 8 | 20000 - 40000 | Mixed (Adults and Peadiatric) | 72 | NICE NG143 |
| Worthing Hospital (WOR) | 10 | 20000 - 40000 | Mixed (Adults and Peadiatric) | 42 | NICE NG143 |

## Table S2: Sensitivity analysis for the diagnostic performance of NICE, BSAC, Aronson rule and AAP with CRP or PCT for IBI in infants ≤ 90 days with main analysis, imputed data and exclusions.

AAP- American Academy of Pediatrics, BSAC- British Society for Antimicrobial Chemotherapy, CDA- Clinical decision aid, CI- Confidence interval, CRP- C-reactive protein, IBI- invasive bacterial infection, NICE-National Institute for Health and Care Excellence, NPV- Negative predictive value, PCT- procalcitonin, PPV- Positive predictive value

Table S2a) Imputed dataset

Total cohort N– 1821 (IBI 67 (3.7%)

| **CDA** | **Low risk** | **IBI in low risk** | **Sensitivity**  **(95% CI)** | **Specificity**  **(95% CI)** | **PPV**  **(95% CI)** | **NPV**  **(95% CI)** |
| --- | --- | --- | --- | --- | --- | --- |
| **NICE NG143** | 466 (26) | 5 (1.1) | 0.93 (0.83 – 0.98) | 0.26 (0.24 – 0.28) | 0.05 (0.04 – 0.05) | 0.99 (0.98 – 1.00) |
| **BSAC** | 329 (18) | 1 (0.3) | 0.99 (0.92 – 1.00) | 0.19 (0.17 – 0.21) | 0.04 (0.04 – 0.05) | 1.00 (0.98 – 1.00) |
| **Aronson rule** | 481 (26) | 7 (1.5) | 0.90 (0.80 – 0.96) | 0.27 (0.25 – 0.29) | 0.05 (0.04 – 0.05) | 0.99 (0.97 – 0.99) |
| **AAP-CRP** | 350 (19) | 1 (0.3) | 0.99 (0.92 – 1.00) | 0.20 (0.18 – 0.22) | 0.05 (0.04 – 0.05) | 1.00 (0.98 – 1.00) |

Table S2b) No bloods (WCC or CRP or ANC) excluded

Total cohort of those who had no WCC or ANC or CRP available is N-1527, IBI rate 4.1% (62/1527)

| **CDA** | **Low risk** | **IBI in low risk** | **Sensitivity**  **(95% CI)** | **Specificity**  **(95% CI)** | **PPV**  **(95% CI)** | **NPV**  **(95% CI)** |
| --- | --- | --- | --- | --- | --- | --- |
| **NICE NG143** | 350 (25) | 5 (1.4) | 0.92 (0.82 – 0.97) | 0.24 (0.21 – 0.26) | 0.05 (0.04 – 0.06) | 0.99 (0.97 – 1.00) |
| **BSAC** | 248 (16) | 1 (0.4) | 0.98 (0.91 – 1.00) | 0.17 (0.15 – 0.19) | 0.05 (0.04 – 0.06) | 1.00 (0.98– 1.00) |
| **Aronson rule** | 381 (25) | 7 (1.8) | 0.89 (0.78 – 0.95) | 0.26 (0.23 – 0.28) | 0.05 (0.04 – 0.06) | 0.98 (0.96 – 0.99) |
| **AAP-CRP** | 271 (18) | 1 (0.4) | 0.98 (0.91 – 1.00) | 0.18 (0.16 – 0.21) | 0.05 (0.04 – 0.06) | 1.00 (0.98 – 1.00) |

Table S2c) Comorbidities excluded

Population N-1561. IBI rate when comorbidities excluded is 3.5% (n-54).

| **CDA** | **Low risk** | **IBI in low risk** | **Sensitivity**  **(95% CI)** | **Specificity**  **(95% CI)** | **PPV**  **(95% CI)** | **NPV**  **(95% CI)** |
| --- | --- | --- | --- | --- | --- | --- |
| **NICE NG143** | 419 (30) | 5 (1.1) | 0.91 (0.80 – 0.97) | 0.27 (0.25 – 0.30) | 0.04 (0.03 – 0.06) | 0.99 (0.97 – 1.00) |
| **BSAC** | 314 (20) | 1 (0.3) | 0.98 (0.90 – 1.00) | 0.21 (0.19 – 0.23) | 0.04 (0.03 – 0.06) | 1.00 (0.98 – 1.00) |
| **Aronson rule** | 448 (29) | 5 (1.1) | 0.91 (0.80 – 0.97) | 0.29 (0.27 – 0.32) | 0.04 (0.03 – 0.06) | 0.99 (0.97 – 1.00) |
| **AAP-CRP** | 347 (22) | 1 (0.3) | 0.98 (0.90 – 1.00) | 0.23 (0.21 – 0.25) | 0.04 (0.03 – 0.06) | 1.00 (0.98 – 1.00) |

Table S2d) Diagnostic performance of AAP with CRP and AAP with PCT for IBI in infants < 90 days.

Population N-466 (with PCT available) IBI rate is 5.4% (n-25)

| **CDA** | **Low risk**  **n (%)** | **IBI in low risk (n (%)** | **Sensitivity**  **(95% CI)** | **Specificity**  **(95% CI)** | **PPV**  **(95% CI)** | **NPV**  **(95% CI)** | **Mean cost per patient (bootstrapped 95% CI)** |
| --- | --- | --- | --- | --- | --- | --- | --- |
| **AAP-PCT+ANC** | 69  (15) | 1 (1.4) | 0.96 (0.80 -1.00) | 0.15 (0.12 – 0.19) | 0.06 (0.04 – 0.09) | 0.99 (0.92 – 1.00) | £1,396 (£1,318 - £1,479) |
| **AAP-CRP +ANC and Temp** | 71 (15) | 0 (0) | 1.00 (0.86 – 1.00) | 0.16 (0.13 – 0.20) | 0.05 (0.04 – 0.08) | 1.00 (0.95 – 1.00) | £1,278 (£1,236 - £1,325) |

## Table S3: Mcnemar test with Bonferroni correction for CDA main analysis, imputation and with exclusions.

AAP- American Academy of Pediatrics, ANC- Absolute neutrophil count, BSAC- British Society for Antimicrobial Chemotherapy, CDA- Clinical decision aid, CI- Confidence interval, CRP- C-reactive protein, IBI- invasive bacterial infection, NICE-National Institute for Health and Care Excellence, PCT-procalcitonin, WCC- White cell count

Table S3a) Main Analysis

Mcnamer test for all 4 CDAs comparing sensitivity and specificity (p-values) main analysis.

| CDAs | NICE NG143 | BSAC | Aronson rule | NICE NG143 | BSAC | Aronson rule |
| --- | --- | --- | --- | --- | --- | --- |
|  | **Sensitivity** | | | **Specificity** | | |
| BSAC | 0.221 | - | - | <0.001 | - | - |
| Aronson rule | 0.752 | 0.077 | - | 0.115 | <0.001 | - |
| AAP (CRP) | 0.134 | 1.000 | 0.077 | <0.001 | <0.001 | <0.001 |

Table S3b) Imputed dataset

Mcnamer test for all 4 CDAs comparing sensitivity and specificity (p-values) imputation analysis.

| CDAs | NICE NG143 | BSAC | Aronson rule | NICE NG143 | BSAC | Aronson rule |
| --- | --- | --- | --- | --- | --- | --- |
|  | **Sensitivity** | | | **Specificity** | | |
| BSAC | 1.000 | - | - | <0.001 | - | - |
| Aronson rule | 1.000 | 0.463 | - | 1.000 | <0.001 | - |
| AAP (CRP) | 0.802 | 1.000 | 0.463 | <0.001 | 0.744 | <0.001 |

Table S3c) No bloods (WCC or ANC or CRP available) excluded.

Mcnamer test for all 4 CDAs comparing sensitivity and specificity (p-values) no bloods excluded.

| CDAs | NICE NG143 | BSAC | Aronson rule | NICE NG143 | BSAC | Aronson rule |
| --- | --- | --- | --- | --- | --- | --- |
|  | **Sensitivity** | | | **Specificity** | | |
| BSAC | 1.000 | - | - | <0.001 | - | - |
| Aronson rule | 1.000 | 0.463 | - | 1.000 | <0.001 | - |
| AAP (CRP) | 0.802 | 1.000 | 0.463 | <0.001 | 0.350 | <0.001 |

Table S3d) Comorbidities excluded.

Mcnamer test for all 4 CDAs comparing sensitivity (p-values) no bloods excluded.

| CDAs | NICE NG143 | BSAC | Aronson rule | NICE NG143 | BSAC | Aronson rule |
| --- | --- | --- | --- | --- | --- | --- |
|  | **Sensitivity** | | | **Specificity** | | |
| BSAC | 1.000 | - | - | <0.001 | - | - |
| Aronson rule | 1.000 | 1.000 | - | 1.000 | <0.001 | - |
| AAP (CRP) | 0.802 | 1.000 | 1.000 | <0.001 | 0.055 | <0.001 |

Table S3e) PCT cohort

Mcnamer test for all 2 AAP CDAs comparing sensitivity (p-values).

| CDAs | AAP CRP | AAP CRP |
| --- | --- | --- |
|  | **Sensitivity** | **Specificity** |
| AAP PCT | 1.000 | 0.690 |

## Table S4: Stepwise binary logistic regression to predict IBI

IBI- Invasive bacterial infection, CI- Confidence interval

| Variable | Odds ratio | CI: 95% | P value |
| --- | --- | --- | --- |
| Clinician opinion of IBI (Likely) | 4.92 | 2.04 – 11.34 | <0.001 |
| Unwell contacts at home or nursery (No) | 4.52 | 1.75 – 15.41 | <0.001 |
| Rash present (No) | 4.44 | 1.31 – 27.69 | 0.044 |
| Coryzal or cough present (No) | 4.06 | 1.67 – 12.16 | 0.005 |

## Table S5. Infants with IBI misclassified by the CDAs (NICE, BSAC, Aronson and AAP-CRP)

AAP- American Academy of Pediatrics, BSAC- British Society for Antimicrobial Chemotherapy, CDA- Clinical decision aid, CRP- C-reactive protein, F- Female, IBI- invasive bacterial infection, M- Male, NICE-National Institute for Health and Care Excellence

| Age days | Comorbidities | Sex | hours from fever onset 2 | Temperature recorded in ED | Unwell appearing | Neutrophil count (x 10*9/L) | C-Reactive protein (mg/L) | Positive urinalysis | Organism (Bacteraemia) | Organism (Meningitis) | NICENG143 | BSAC | Aronson rule | AAP-CRP |
| --- | --- | --- | --- | --- | --- | --- | --- | --- | --- | --- | --- | --- | --- | --- |
| 44 | No | M | 2 (>6hrs) | 38 | No | 2.29 | 49 | No | Staphylococcus aureus |  | Low risk | High risk | High risk | High risk |
| 40 | No | M | 1 (<6hrs) | 36 | No | 3.4 | 55 | No | Escherichia coli |  | Low risk | High risk | Low risk | High risk |
| 74 | No | M | 2 (>6hrs) | 36.6 | No | 3.8 | 49 | Yes | Escherichia coli |  | Low risk | High risk | High risk | High risk |
| 58 | No | F | 1 (<6hrs) | 36.9 | No | 4.4 | 18 | Yes |  | Enterobacter cloacae | Low risk | High risk | High risk | Low risk |
| 36 | No | F | 2 (>6hrs) | 37.4 | No | 3.6 | 68 | Yes | Escherichia coli |  | Low risk | High risk | High risk | High risk |
| 36 | No | M | 1 (<6hrs) | 36.6 | No | 8.9 | 2 | No | Staphylococcus aureus |  | High risk | Low risk | High risk | High risk |
| 17 | No | F | 1 (<6hrs) | 37.9 | Yes | 4.2 | 2 | No | Staphylococcus aureus |  | High risk | High risk | Low risk | High risk |
| 47 | Yes | M | 1 (<6hrs) | 37.3 | Yes | 0.46 | 5 | No | Streptococcus agalactiae (GBS) |  | High risk | High risk | Low risk | High risk |
| 14 | No | M | 2 (>6hrs) | 37.7 | Yes | 2.34 | 65 | No | Escherichia coli |  | High risk | High risk | Low risk | High risk |
| 61 | Yes | M | 2 (>6hrs) | 36.7 | Yes | 1.9 | 326.1 | No | Escherichia coli (Klebsiella pneumonia) |  | High risk | High risk | Low risk | High risk |
| 17 | No | M | 1 (<6hrs) | 37.2 | No | 1.3 | 13 | No | Streptococcus agalactiae (GBS) |  | High risk | High risk | Low risk | High risk |
| 15 | No | M | 2 (>6hrs) | 36.6 | Yes | 2.7 | 298 | No | Enterobacter asburiae |  | High risk | High risk | Low risk | High risk |
| 33 | Yes | M | 2 (>6hrs) | 37.1 | Yes | 4.5 | 21 | Yes | Escherichia coli |  | High risk | High risk | High risk | High risk |

## Table S6: Infant misclassified by AAP-PCT CDA in cohort with PCT available (n-466)

AAP- American Academy of Pediatrics, CDA- Clinical decision aid, CRP- C-reactive protein, M- Male, PCT- Procalcitionin

| Age days | Comorbidities | Sex | hours from fever onset 2 | Temperature recorded in ED | Unwell appearing | Neutrophil count (x 10*9/L) | C-Reactive protein (mg/L) | Positive urinalysis | Organism (Bacteraemia) | Organism Meningitis | AAP-PCT | AAP-CRP |
| --- | --- | --- | --- | --- | --- | --- | --- | --- | --- | --- | --- | --- |
| 74 | No | M | 2 (>6hrs) | 36.6 | No | 3.8 | 49 | Yes | Escherichia coli | - | Low risk | High risk |

## Table S7: Unit costs and sources

CC- Complication and comorbidity, CII- Cost Inflation Index ED- Emergency department, GBP- Great British Pound sterling, NHS- National Health Service, NICE-National Institute for Health and Care Excellence, PCT- Procalcitonin

| **Resource item** | **Unit cost (GBP 2022/23 prices)** | **Source** |
| --- | --- | --- |
| ***ED attendance*** | | |
| ED attendance with investigation including lumbar puncture, administration of antibiotics and admission to hospital (Emergency Medicine, Any Investigation with Category 5 Treatment) | £420 per spell | NHS England and NHS Improvement (2022) |
| ED attendance with investigation, administration of antibiotics and admission to hospital (Emergency Medicine, Category 3 Investigation with Category 4 Treatment) | £420 per spell | NHS England and NHS Improvement (2022) |
| ED attendance with investigation and admission to hospital (Emergency Medicine, Category 3 Investigation with Category 1-3 Treatment) | £295 per spell | NHS England and NHS Improvement (2022) |
| ED attendance with investigation and discharged home directly (Emergency Medicine, Category 2 Investigation with Category 2 Treatment) | £189 per spell | NHS England and NHS Improvement (2022) |
| PCT test | £16.71 per test | NICE (2015) DG18 and uplifted to 2022/23 prices using the NHS CII pay and prices (Jones et al., 2024) |
| ***Hospital admission*** | | |
| Hospital admission for paediatric fever of unknown origin with CC score 1-2, as non-elective inpatient – short stay (trim point 5 days) | £839 per spell | NHS England and NHS Improvement (2022) |
| Hospital admission for paediatric major infections with CC score 2-4, as non-elective inpatient – long stay (trim point 10 days) | £2,888 per spell | NHS England and NHS Improvement (2022) |
| Excess bed day | £464 per day | NHS England and NHS Improvement (2022) |
| ***Follow-up health service*** | | |
| Ambulatory care, as non-admitted face-to-face attendance, follow-up | £166 per spell | NHS England and NHS Improvement (2022) |
|  | | |

## Table S8: Participating sites and investigators (Paediatric Emergency Research in the UK and Ireland (PERUKI)

| Airedale NHS Foundation Trust | Phillipa Rawling, Amy Kitching, Firyuza Yunusova |
| --- | --- |
| Alder Hey Children's Hospital NHS Foundation Trust | Shrouk Messahel, Sarah Siner, Stephen Fletcher, Karen Geoghegan, Laura Tremarco |
| Bradford Teaching Hospitals NHS Trust | Suvradeep Basu, Rachel Swingler, Cristina Hearnshaw |
| Bristol Royal Hospital for Children | Mark Lyttle, Tracey Bingham, David Hopgood, Ruth Bonsor, Cath Jones |
| Cardiff and Vale University Health Board | Jordan Evans, Marianne Jenkins, Heba Mansour, Jennifer Muller, Anna de Carteret, Rhian Thomas-Turner |
| Children's Health Ireland at Crumlin | Michael Barrett, Maire Bourke, John Coveney, Cathal De Buitleir, Heather Grace, Maja Kuhar, Kristi Constantinou, Dani Hall, Maria Angela Garcia Cadena, Carol Blackburn, Stuart Kinane, Mishra Ashmin, Kate Bruton, Erica Lahoud, Santhiska Pather, Luke Fletcher, Karl Kavanagh |
| Countess of Chester NHS Foundation Trust | Murthy Saladi, Caroline Burchett, Sarah de-Beger, Jenny Loughnane |
| Imperial College Healthcare NHS Trust | Ruud G. Nijman, Ian K. Maconochie |
| King's College Hospital | Fleur Cantle, Clare Finney, Andrew Dodd |
| Leeds Teaching Hospitals NHS Trust | Alice Downes, Joanne Anderson, Jane Muir, Collette Spencer |
| Mid Yorkshire Teaching Trust | Victoria Hemming, Gail Castle, Dawn Athorn |
| Musgrove Park Hospital | Esther Wilson, Wayne Battishill, Charmaine Shovelton, |
| Newham University Hospital, Barts Health NHS Trust | Bahadur Anjum, Ivone.Lancoma-Malcolm, Susan Liebeschuetz, Farah Sheikh, Rakesh Ravi, Evangelia Loannidou, Grace Chin Lay Tan, Marvee Abro, Haleema Nuwera |
| North Tees and Hartlepool NHS Trust | George Simpson, Dawn Egginton |
| North West Anglia NHS Foundation Trust | Katharine McDevitt, Alys Capell, Helen Wilson |
| Poole Hospital | Edward Andrews, Sarah Mills, Lucy Rubick, Amy Roff, Oana Langston, Francesca Diaz, Jemma Parratt |
| Portsmouth Hospitals University NHS Trust | Alan Charters, Simon Birch, Emma McCloud, Emma Helyer, Zoe Daly, Andrew Gribbin, Halimah Tasnim, Hannah Paul, Sharon Glaysher |
| Royal Alexandra Children's Hospital, Brighton | Emily Walton, Vivien Richmond, Melanie Ranaweera, Michela Gandolfo |
| Royal Belfast Hospital for Sick Children | Thomas Waterfield, Kathryn Wilson |
| Royal Berkshire NHS Foundation Trust | Manish Thakker, Annie Warrington, Emma Gammin, Shashank Poonam, Sharon Westcar, Egi Krasniqi, Jemima Hepburne-Scott, Eleanor Didier'Serre |
| Royal Cornwall Hospital Trust | Kim Lindsey |
| Royal Hospital for Children & Young People, Edinburgh | Jen Browning, Ashleigh Hegan, Caroline Blackstock, Connor McLaughlin |
| Royal Hospital for Children, Glasgow | Steven Foster, Katherine Longbottom, Annette Frager |
| Royal London Hospital, Barts Health NHS Trust | Rebecca Platt, Raine Astin-Chamberlain, Grace Tunesi, Noemi Caponi, Nimca Omer, Jacqueline Sear, Alison Quinn |
| Sheffield Children's NHS Foundation Trust | Shammi Ramlakhan, Lucine Nahabedian, Julie Morcombe, Kathryn Jones |
| South Tyneside and Sunderland NHS Foundation Trust | Niall Mullen, Louise Fairlie, Gemma Salt |
| The James Cook University Hospital | Jonathan Lane, Arshid Murad, Sophie Rodger |
| University Hospital Southampton NHS Foundation Trust | David James, Emy Van Der Harg, Susan Burke, Louise Anthony, Amber Cook |
| University Hospitals Leicester | Damian Roland, Elsa Mathew, Samantha Hunt, Christopher Dadnam |
| Watford General Hospital | Jason Palman, Kat Priddis, Chiara Ellis |
| Whipps Cross Hospital, Barts Health NHS Trust | Amutha Anpananthar, John Ho, Nosheen Khalid, Theresa Simangan, Aimee-Louise Hamlin, Kevin Samuels, Madiha Islam |
| Whiston Hospital, Mersey and West Lancashire Teaching Hospitals NHS Trust | Clare O'Leary, Kerri Bowness |
| Whittington Health | Erum Jamall, laura Jeffers, Lily Kirkpatrick, Erin Hart, Hama Baqaeen |
| Worthing hospital, St Richard's hospital Chichester | Claire Meager, Sharon Floyd, Clinton Corin, Frances Blackburn, Erikka Siddall, Sulagna Roy |
